# Identification of candidate enhancers controlling the transcriptome during the formation of interphalangeal joints

**DOI:** 10.1038/s41598-022-16951-4

**Published:** 2022-07-27

**Authors:** Karol Nowosad, Rutger W. W. Brouwer, Adrian Odrzywolski, Anne L. Korporaal, Bartłomiej Gielniewski, Bartosz Wojtaś, Wilfred F. J. van IJcken, Frank Grosveld, Danny Huylebroeck, Przemko Tylzanowski

**Affiliations:** 1 Department of Cell Biology, Erasmus University Medical Center, 3015 CN Rotterdam, The Netherlands; 2 Department of Biomedical Sciences, Laboratory of Molecular Genetics, Medical University of Lublin, Lublin, Poland; 3 The Postgraduate School of Molecular Medicine, Medical University of Warsaw, Warsaw, Poland; 4 Center for Biomics-Genomics, Erasmus University Medical Center, 3015 CN Rotterdam, The Netherlands; 5 Department of Biochemistry and Molecular Biology, Medical University of Lublin, Lublin, Poland; 6 Department of Human Genetics, KU Leuven, 3000 Leuven, Belgium; 7 Laboratory of Molecular Neurobiology, Nencki Institute of Experimental Biology of the Polish Academy of Sciences, Warsaw, Poland; 8 Laboratory of Sequencing, Nencki Institute of Experimental Biology of the Polish Academy of Sciences, Warsaw, Poland; 9 Department of Development and Regeneration, Skeletal Biology and Engineering Research Center, KU Leuven, 3000 Leuven, Belgium

**Keywords:** Cartilage development, Transcriptomics, Regulatory networks

## Abstract

The formation of the synovial joint begins with the visible emergence of a stripe of densely packed mesenchymal cells located between distal ends of the developing skeletal anlagen called the interzone. Recently the transcriptome of the early synovial joint was reported. Knowledge about enhancers would complement these data and lead to a better understanding of the control of gene transcription at the onset of joint development. Using ChIP-sequencing we have mapped the H3-signatures H3K27ac and H3K4me1 to locate regulatory elements specific for the interzone and adjacent phalange, respectively. This one-stage atlas of candidate enhancers (CEs) was used to map the association between these respective joint tissue specific CEs and biological processes. Subsequently, integrative analysis of transcriptomic data and CEs identified new putative regulatory elements of genes expressed in interzone (e.g., *GDF5*, *BMP2* and *DACT2*) and phalange (e.g., *MATN1*, *HAPLN1* and *SNAI1*). We also linked such CEs to genes known as crucial in synovial joint hypermobility and osteoarthritis, as well as phalange malformations. These analyses show that the CE atlas can serve as resource for identifying, and as starting point for experimentally validating, putative disease-causing genomic regulatory regions in patients with synovial joint dysfunctions and/or phalange disorders, and enhancer-controlled synovial joint and phalange formation.

## Introduction

Synovial joints, organs present at the articular ends of long bones, are essential for vertebrate mobility. They comprise of articular cartilage, synovium, ligaments and the synovium capsule^1^. Due to their function, the joints are frequently exposed to mechanical stress and thus prone to injuries. Congenital malformations and a number of diseases affect joint structure, thereby causing a decrease of joint functionality. For instance, misexpression of *PITX1* caused by enhancer adoption results in dysplastic elbow joints in Liebenberg syndrome (OMIM #186550)^2^, a homozygous mutation in *IMPAD1* leads to chondrodysplasia with joint dislocations (OMIM #614078)^3^, and loss of *EXOC6B* causes joint dislocations and defects in joint mobility, characteristic for patients with spondylo-epimetaphyseal dysplasia with joint laxity, type 3 (OMIM #618395)^4^. Osteoarthritis (OA) is the most prevalent synovial joint disease affecting adults^5^. Typical in OA is the progressive degeneration of articular cartilage and accompanying subchondral bone sclerosis, joint space narrowing, osteophyte formation and the variable degree of synovium inflammation^6^, eventually causing joint destruction. The latter frequently needs intervention by joint replacement^7^.

A comprehensive understanding of gene regulatory networks (GRNs) orchestrating synovial joint formation will contribute to the understanding of both healthy and pathological processes taking place in this organ. This knowledge will also help in the development of novel cell and/or gene-based strategies for treatment of injured articular cartilage within the developmental engineering paradigm^8^. The first morphologically distinguishable event in joint development is the formation of interzones, with distinct progenitor cells giving rise to the majority of articular tissues^9^. The condensing cartilage anlage, at the locations of future joints, undergoes several rounds of cell proliferation^10^. The discovery that the influx of cells from the outside of the interzone contributes to overall increased interzone cell density, points to an important mechanism in interzone formation^11,12^. At the same time, *SOX9* [a member of Sry family of transcription factors (TFs)] expression becomes repressed, arresting the chondrogenic program and allowing the interzone to form^13^. The formed joint interzone comprises of two layers of cells, named the outer and intermediate interzone layers, with differentially expressed genes (DEGs), including *COL2A1* (encoding α(II)-collagen) and *MATN1* (Matrilin-1), which have higher expression in the outer interzone, and *GDF5* (Growth and Differentiation Factor-5, a ligand of the BMP subgroup of the TGFβ family) with higher mRNA level in the intermediate interzone^14^. These layers will contact the ends of future bones, while an inner cell layer of yet to be defined function is also present.

Cells within the cartilage anlage change their phenotype progressively from round to columnar, pre- and eventually hypertrophic chondrocytes, contributing to longitudal cartilage and bone growth. Acknowledged molecular markers for the round chondrocytes include the aforementioned *MATN1* and *COL2A1*^15^. Additionally, chondrogenic induction and differentiation is accompanied by the expression of *RUNX2* (encoding a RUNT family TF) followed by the expression of *COL10A1* (α(X)-collagen). The latter is a specific marker gene for hypertrophic chondrocytes, and some of its enhancers have been mapped^16^. It has been suggested that the round chondrocytes may contribute to the articular cartilage, but their contribution to the interzone structures remains unclear and may depend on restricted exposure to BMP and/or WNT signals^11^. Thus, while new knowledge is emerging regarding early stages of joint formation, including interaction with adjacent cartilage, the insight into gene expression control within cells of the joint interzone remains incomplete. Indeed, while numerous reports have described the transcriptome in the synovial joint formation^14,17^, including at single-cell level^18^, relatively little is known about the activity of enhancers during that process.

Enhancers regulate gene transcription mainly in *cis*, within the topologically-associating domains (TADs), where they promote intra-TAD control of transcription of loci by making TFs and co-factors bridge between their bound distal enhancer sites and the promoter-proximal region of the appropriate target gene(s), hence achieving physical proximity^19–21^. Enhancers are associated with histone modification signatures, such as H3K27ac and H3K4me1, and chromatin accessibility, however such biochemical marks may be absent in the so-called hidden enhancers at one or more stages of cell differentiation. In the enhancer-promoter complexes high-affinity binding of co-factors (e.g. histone-acetylation containing P300), TFs and RNA-Pol2 also can be shown^22^. Defects in enhancer function have been linked to limb malformations as well, for example in Hass-type polysyndactyly (OMIM #186200)^23^, split hand/foot malformation (OMIM #183600)^24^, Leri-Weill dyschondrosteosis (OMIM #127300) and Laurin-Sandrow syndrome (OMIM #135750)^25^.

Here, our focus was on identifying candidate enhancers (CEs) based on biochemical H3-profiles active in the joint interzone and adjacent phalange. Next, we developed an atlas of candidate *cis*-regulatory elements at one developmental stage of chick embryos. In combination with the available transcriptomes, this atlas will help in elucidating the molecular mechanisms that control joint interzone formation and/or cause joint disease. We opted for microsurgical dissection of interzone as opposed to using *GDF5*-positive (+) cell selection procedures, because not all cells during early stages of joint formation are convincingly *GDF5*+^12,18^. We identified unique interzone/phalange CEs that are conserved between chicken, mouse and human, and functionally annotated these CEs, followed by integrative analysis of cell-type specific CEs and DEGs. We also associated the CEs with synovial joint and phalange abnormalities, and a higher risk of OA.

## Results

### Microdissection of joint interzones and phalanges

**Figure 1:**
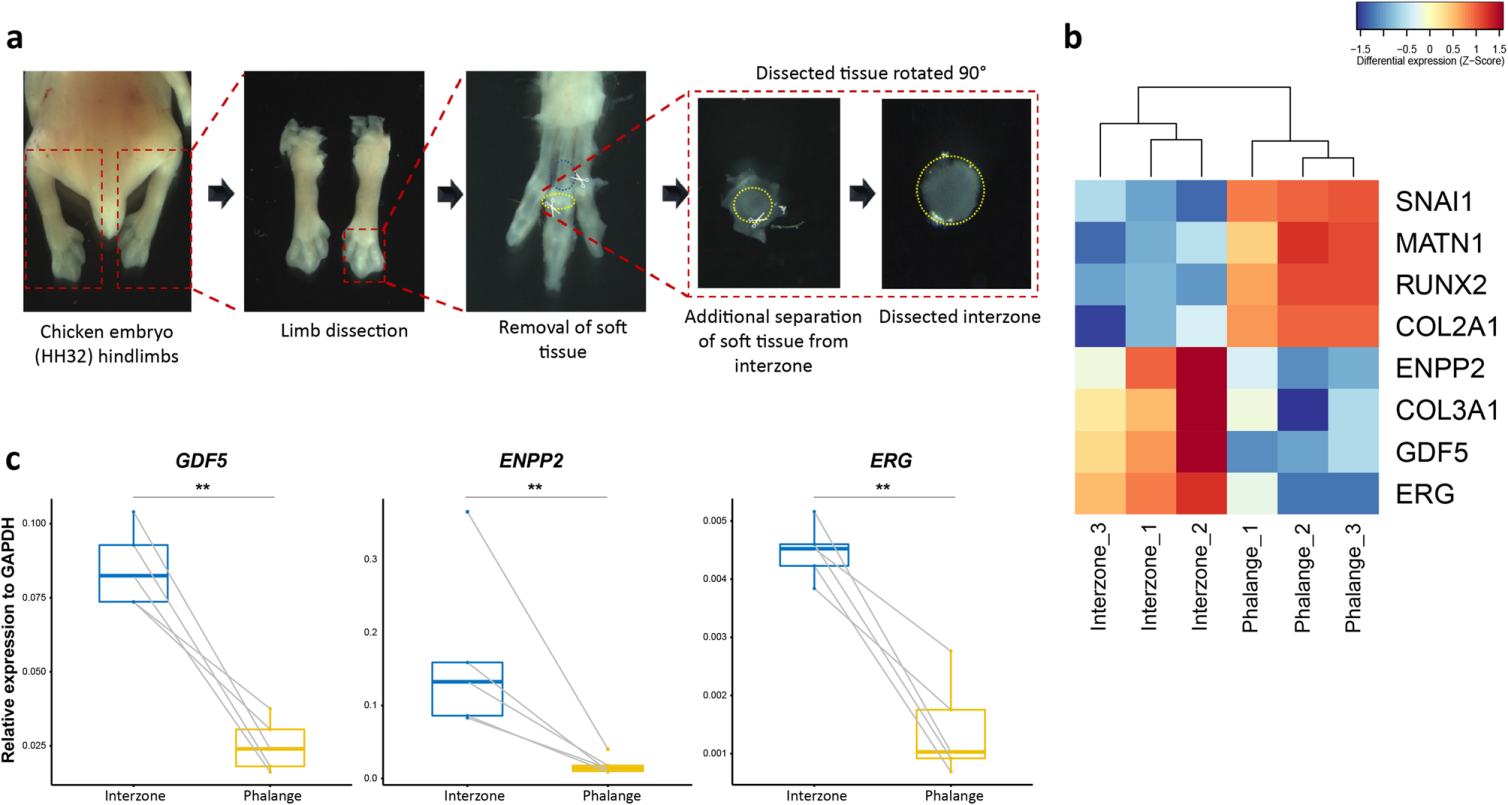
Dissection and transcriptome profiling of joint interzone and phalanges. (**a**) Location and dissection of the interzone and adjacent proximal part of phalange from hindlimb digit 3 of chicken embryo (HH32). The dissection procedure includes separation of hindlimbs, removal of soft tissue from the digits, and subsequent separation of interzone from adjacent phalange. Dissection was performed using Zeiss Stereo Discovery V8 microscope equipped with 0.63 × Plan Apo S Objective Lens and images were taken using ZEISS ZEN 3.2 (blue edition) software https://www.zeiss.com/microscopy/int/products/microscope-software/zen.html. (**b**) Differences in expression of interzone (*ENPP2*, *COL3A1*, *GDF5*, *ERG*) and phalange (*SNAI1*, MATN1, *RUNX2*, *COL2A1*) marker genes based on the RNA-seq data. (**c**) mRNA steady-state level of selected interzone markers (*GDF5*, *ENPP2*, *ERG*) as determined by RT-qPCR (all data were normalized to the expression of *GAPDH*; lines combined the samples isolated from the same embryo; *p < 0.05; **p < 0.01 based on Mann–Whitney–Wilcoxon test).

The interzones and the adjacent proximal part of phalange were dissected from the third digit of the hindlimb of chick embryos (at stage HH32, when the interzone was distinguishable under the microscope; Fig. 1a), and RNA-sequencing (RNA-seq) was performed on the separated tissues (see “Materials and methods”). Subsequently, we analyzed the interzone and phalange RNA-seq datasets using DESeq2. First, we checked whether biological replicates separated according to origin of the tissue. For this, we performed principal component analysis (PCA) revealing that component 1 (PC1) indeed separates interzone from phalange (Supplementary Fig. 1a). Next, to ensure that our datasets fit the DESeq2 model we analyzed the dispersion estimates, showing that our data generates typical pattern of dispersion plot (Supplementary Fig. 1b). Importantly, the curve presented at this plot has low dispersion values for high mean values of normalized counts, and high dispersion values for low mean values of normalized counts, which presents a general relationship between dispersion and gene expression for datasets fitting the DESeq2 model.

Next, we performed gene expression analysis (DEA) to identify key gene players specific either for interzone or adjacent phalange. The DEA with log2FC > 0.5 and p.adj < 0.05 identified 116 upregulated genes in interzone, and 61 genes upregulated in phalange (Supplementary Table 1; Supplementary Fig. 1c). Importantly, this analysis confirmed that the interzone samples had increased mRNA levels of *GDF5*, *ENPP2, COL3A1* and *ERG*, each already known to be expressed in joint interzones. Also, the DEA showed that phalange samples had significantly higher expression of well-described chondrocyte markers, such as *COL2A1*, *MATN1*, *SNAI1* and *RUNX2* (Fig. 1b; Supplementary Table 1). Notably, *COL10A1* mRNA expression was not detected in phalange samples, supporting the notion that the collected phalange regions contain chondrocytes prior to hypertrophy, indicating an early stage of the limb development. Also, interzone samples were not expressing *COL10A1* (except one replicate with ultra-low count numbers, i.e. equal to 1.9, suggesting that this is an artefact, and not the product of gene expression) excluding potential contamination by hypertrophic chondrocytes (Supplementary Table 2). To further validate differential expression of selected interzone marker genes (i.e. *GDF5*, *ENPP2*, *ERG*) we performed RT-qPCR (Fig. 1c), which confirmed our RNA-seq data. Next, we compared gene expression of our identified DEGs with available single cell (sc)RNA-seq data from atlas of synovial joint development^18^. For this purpose, we focused on the Super Cluster 2 (SC2), shown to be composed of two populations: SC2_A, expressing chondrocyte related genes, and SC2_B, expressing interzone markers. Subsequently, we analyzed the subset of 60 genes due to lack of information about the other DEGs in scRNA-seq data. Many of the DEGs identified here presented high expression either in SC2_A, or SC2_B (Supplementary Fig. 2–4). Collectively, these results show that we successfully dissected tissues of interest.

### Atlas of putative enhancers of joint interzone and phalange identifies candidate enhancers involved in the regulation of cell identity

**Figure 2:**
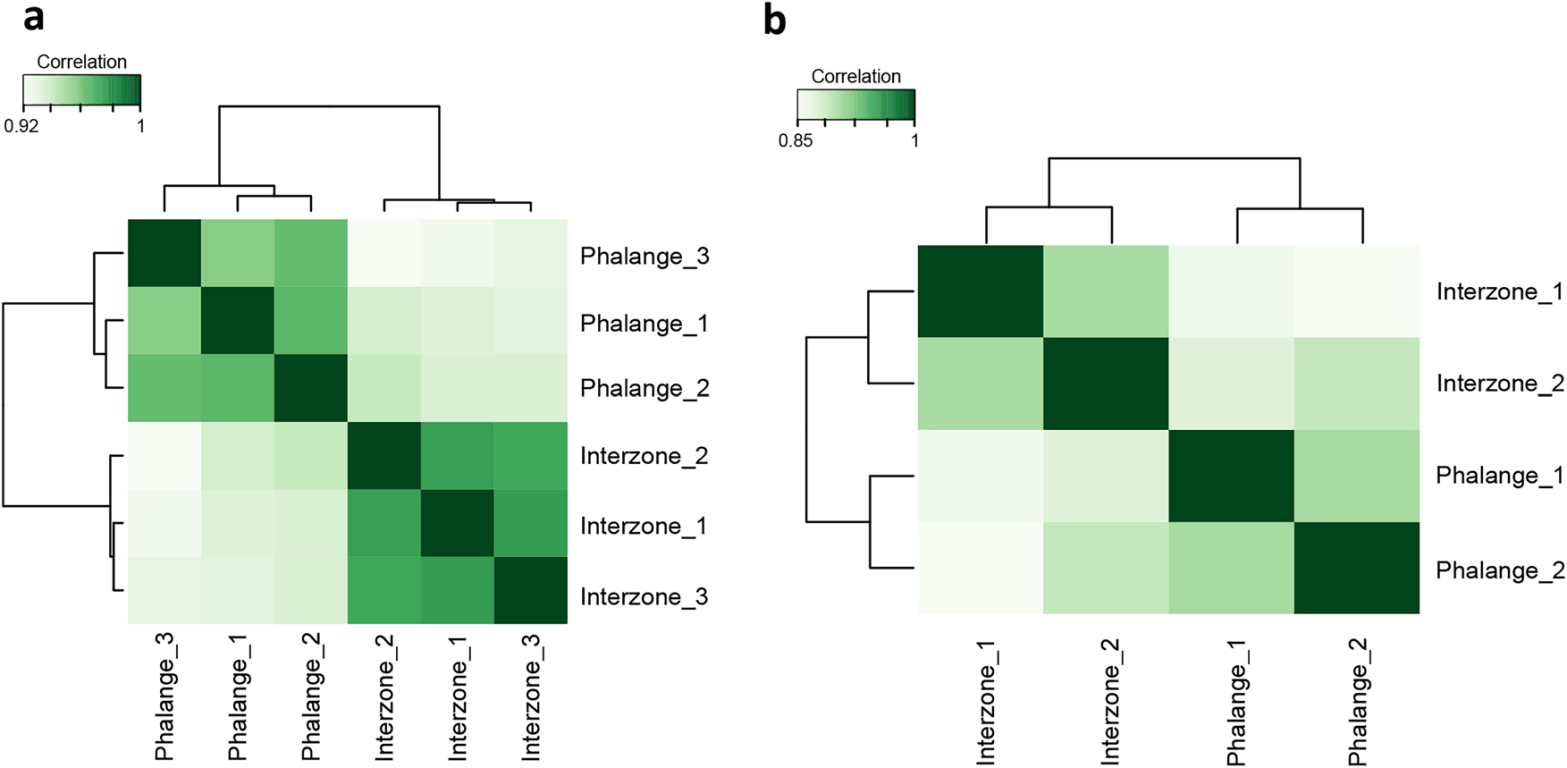
Clustering of genome-wide profiles of histone modifications in prepared interzone and phalange, in particular based on the mapping of H3K27ac and H4K4me1 genomic regions using ChIP-seq. (**a**) Heatmap presenting correlation of interzone and phalange, based on the detection of H3K27ac. (**b**) Heatmap with correlation of interzone and phalange H3Kme1 mapped signatures.

**Figure 3:**
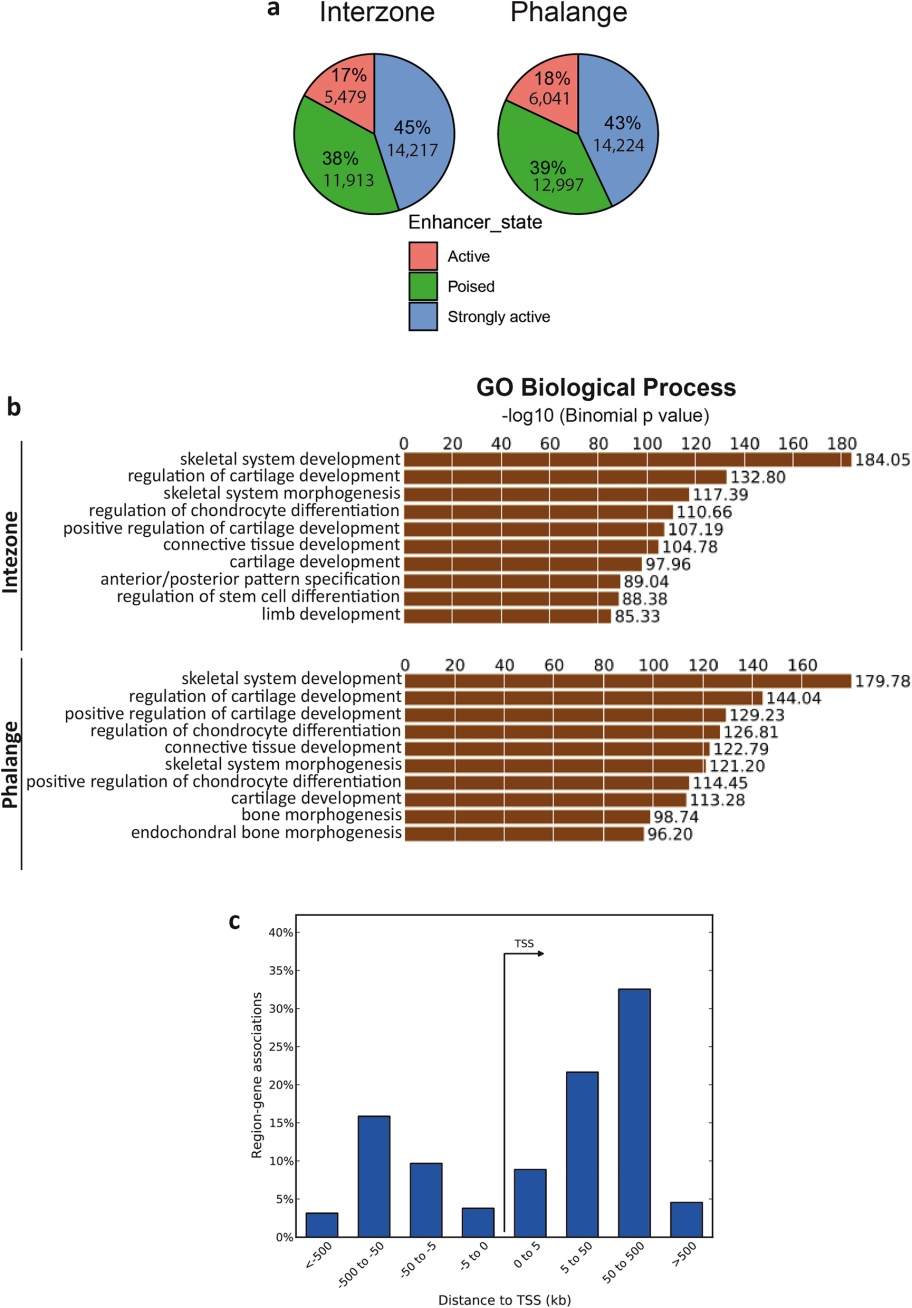
Characterization of the strongly-active CEs. (**a**) Distribution of strongly-active, active and poised candidate enhancers in the interzone and phalange enhancer atlas. (**b**) Biological process GO terms associated with the strongly-active CEs. The functional annotation of CEs was again carried out using GREAT. (**c**) Genomic localization of strongly-active CEs in relation to nearby transcription start site (TSS). The CEs—target gene(s) association and calculation of the distances from the TSS was performed using GREAT.

Next, we mapped the global H3K27ac and H3K4me1 signatures of joint and phalange by chromatin immunoprecipitation followed by next-generation sequencing (ChIP-seq). The unsupervised clustering analysis revealed that interzone and phalange have distinct profiles for both H3K27ac and H3K4me1 (Fig. 2a,b). The mapping of the regions enriched for H3 modifications enabled us to generate a joint/phalange CE atlas and regions enriched for both H3K27ac and H3K4me1 were denoted strongly-active enhancers, for H3K27ac active enhancers, and for H3K4me1 poised enhancers. Since enhancers are often evolutionarily conserved^26^, we decided to select only conserved regions among chick, mouse and human followed by merging nearby genomic intervals (for details see “Material and methods”). Merging of these conserved regions reduces the probability of CEs separation into multiple short sequences and empower the analysis, however, may lead to the generation of CEs containing multiple conserved regions separated by non-conserved genomic blocks. Using this approach, we identified 14,217 strongly-active, 5479 active and 11,913 poised enhancers in the interzone, and 14,224, 6041 and 12,997, respectively, in the phalange (Supplementary Table 3; for their frequency and similar ratios in both samples, see Fig. 3a).

For functional annotation of these CEs we used GREAT, which extracts gene ontology (GO) terms linked to biological processes^27^. The denoted strongly-active enhancers associated with cartilage and skeletal development (Fig. 3b), whereas active and poised enhancers mostly linked to general cell functions or processes not specific for skeletal development (Supplementary Fig. 5). These results suggest that, among all CEs, only the strongly-active enhancers are associated with genes involved in regulation of processes crucial for limb development. Therefore, we hypothesized that strongly-active enhancers play important role in cell-type specific biological processes.

**Figure 4:**
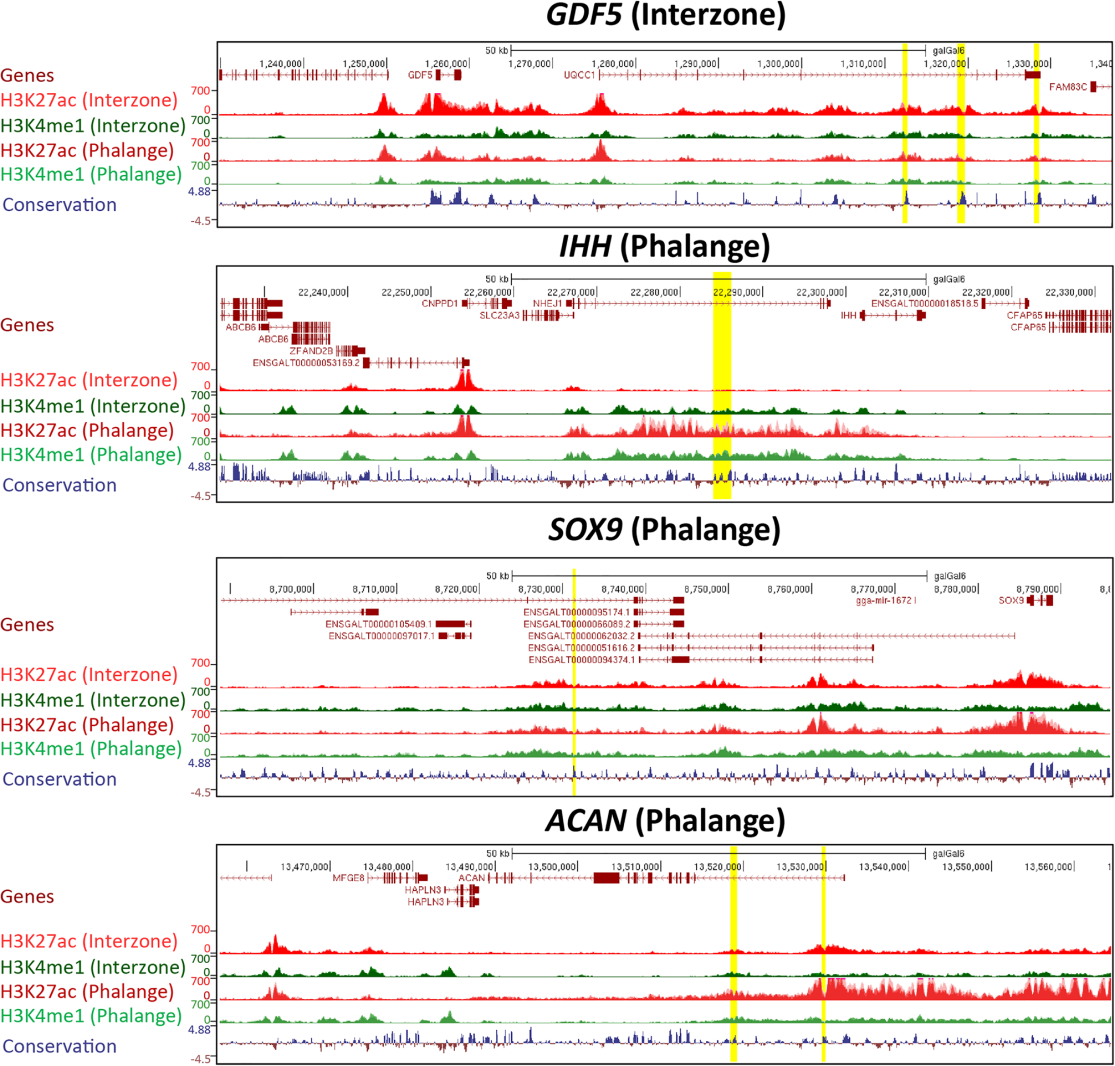
Examples of functionally validated enhancers characterized in the literature. For the typical four loci shown, the H3K27ac enrichment track is marked in red, the H3K4me1 enrichment in green, and the conservation track in blue (together with gene structure information in brown). Regions marked by yellow present functionally validated enhancers described in^28–31^. The same enhancers have been identified as strongly-active enhancers in our Enhancer Atlas.

Association of strongly-active candidate enhancers with cell-lineage specific processes prompted us to focus on strongly-active CEs. These CEs are typically located > 5 kb away from the respective transcription start site (TSS) (Fig. 3c). We could confirm characterized enhancers of well-studied loci (Fig. 4), specifically those expressed in interzone (e.g., *GDF5*)^28^ or chondrocytes (e.g., phalangeal *IHH*, *SOX9, ACAN*)^29–31^. In parallel, and further validating our in silico selection approach, we extended the analysis by using Vista Enhancer Browser dataset^32^, leading to identification of 257 enhancers (Supplementary Table 4), which have been functionally validated during embryogenesis. The enhancers from the Vista Enhancer Browser dataset were tested at E11.5 in mouse. Using our atlas, we showed that these 257 enhancers also present marks of active enhancers at later developmental stage (HH32, an equivalent of E14.5 in mouse) and also present conserved activity in chicken. A majority of them (203/257) have been defined as strongly-active CEs in both interzone and phalange (for illustration of 6 of these, see Supplementary Fig. 6).

**Figure 5:**
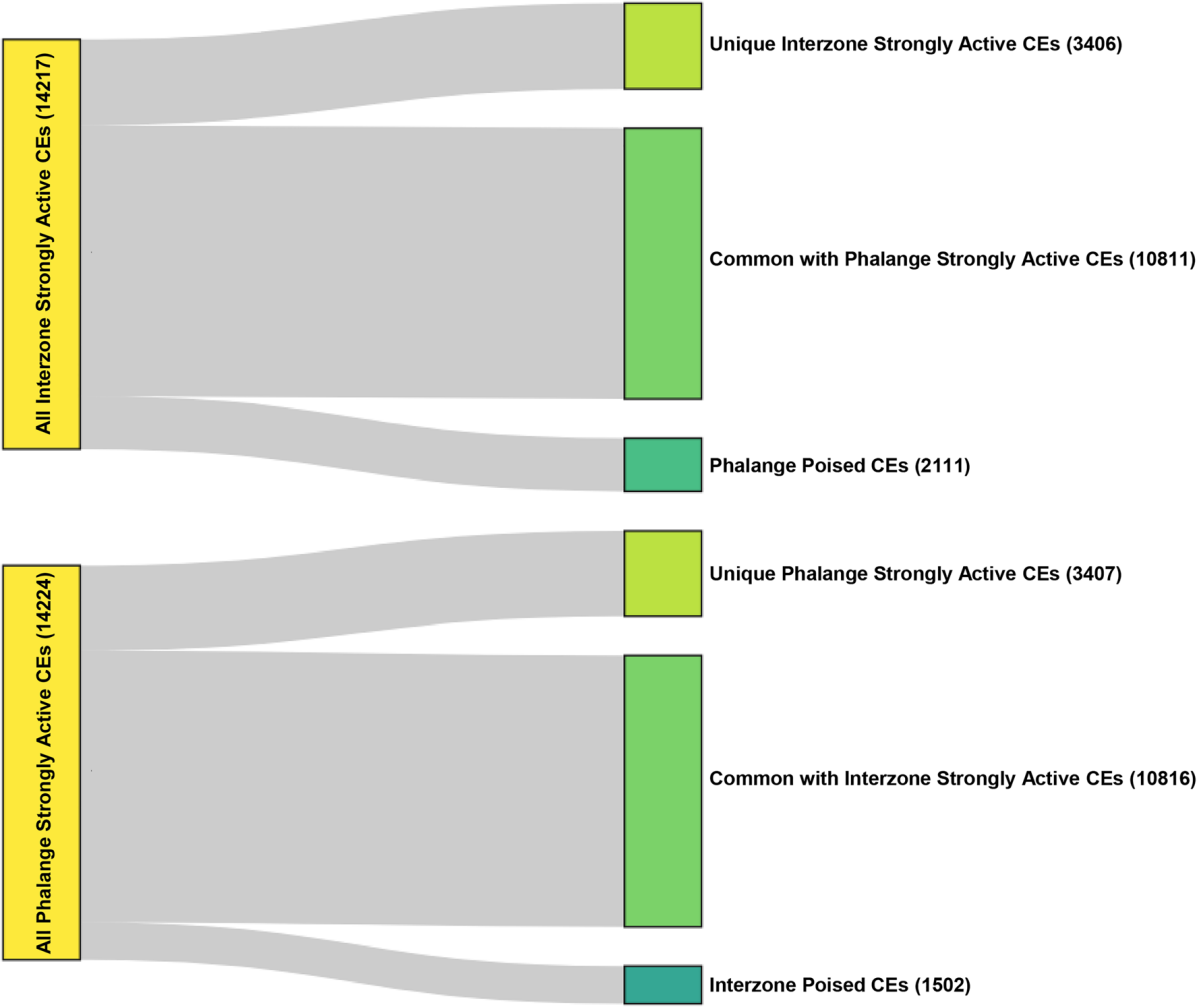
Analysis of unique CEs and CEs with different enhancer states. Visualization of all CEs with a subset of unique CEs and a subset of CEs which change the state between tissues.

To further characterize tissue-specific strongly-active CEs in the developing interzone and phalange, we selected the mutually exclusive strongly-active enhancers, yielding 3406 CEs (out of the aforementioned 14,217 in total) unique for interzone and 3407 (out of 14,224) for phalange (Supplementary Table 5, Fig. 5). GREAT linked many of such interzone-specific CEs to mesenchymal cell differentiation, and regulation of transmembrane receptor protein serine/threonine kinase signaling (Supplementary Fig. 7a, top panel). In contrast, CEs exclusive for phalange retrieved GO terms including chondrocyte differentiation and endochondral bone morphogenesis (Supplementary Fig. 7b, top panel).

Next, we investigated whether the change of enhancer state from strongly-active to poised would be relevant for the regulation of tissue-specific genes. Indeed, 2111 changes occurred with strongly-active CEs (out of the aforementioned identified 14,217 in total) in interzone and were found poised in phalange; in comparison, 1502 changes occurred with strongly-active CEs (out of 14,224 in total) in phalange and were found poised in interzone (Fig. 5; Supplementary Table 6). Strikingly, the interzone strongly-active enhancers that are poised in phalange were found to associate with regulation of transmembrane receptor protein serine/threonine kinase signaling (Supplementary Fig. 7a; bottom panel), which is consistent with our functional annotation of interzone-specific CEs. In contrast, phalange strongly-active enhancers that are poised in interzone linked to positive regulation of cartilage differentiation (Supplementary Fig. 7b; bottom panel).

### Transcription factor binding at strongly-active CEs of developing interzone and phalange

Enhancers contain multiple TF binding-sites (TFBSs), involved in enhancer activation. The formation of TFBSs into clusters within enhancer regions allows enhancers to be bound by a set of tissue-specific TFs, and consequently be regulated in a spatio-temporal manner. Therefore, to predict the binding of TFs with function/s in limb development (including synovial joint development), we performed motif enrichment analysis of interzone and phalange strongly-active CEs identified in this study.

The analysis of TFBSs using HOMER^33^ revealed that strongly-active CEs from both tissues are indeed enriched in TFs motifs pivotal for limb development (Supplementary Tables 7 and 8). A majority of identified motifs was common for both interzone and phalange CEs. Importantly, we identified that CEs were enriched in the motif belonging to P300, a co-factor associated with active enhancers. There was also a match to PITX1, a TF crucial for hindlimb identity^34^, as well as a match to *ERG*, a TF involved in synovial joint formation^35^ and OA susceptibility^36^, and to a motif of *HOXD13*, a TF important for phalange formation and disease^37^.

Next, we characterized tissue-specific TFs, which may regulate interzone and phalange CEs and be responsible for establishment of cell identity. For this purpose, we performed motif enrichment analysis using mutually exclusive strongly-active enhancers CEs specific for interzone and phalange (Supplementary Tables 9 and 10). For instance, we identified that phalange-specific CEs contain exclusively enrichment of *RUNX2* motifs in comparison to interzone-unique CEs. Importantly, *RUNX2* was identified as differentially expressed gene (log2FC = 2.5; p.adj = 4.5e^−23^) in our interzone and phalange RNA-seq datasets (Fig. 1b). Collectively, the motif analysis showed that identified CEs are enriched in the motifs of TFs, which are important for development of synovial joint and phalange, as well as matching with our RNA-seq data also.

### Integrative analysis of DEGs and CEs

**Figure 6:**
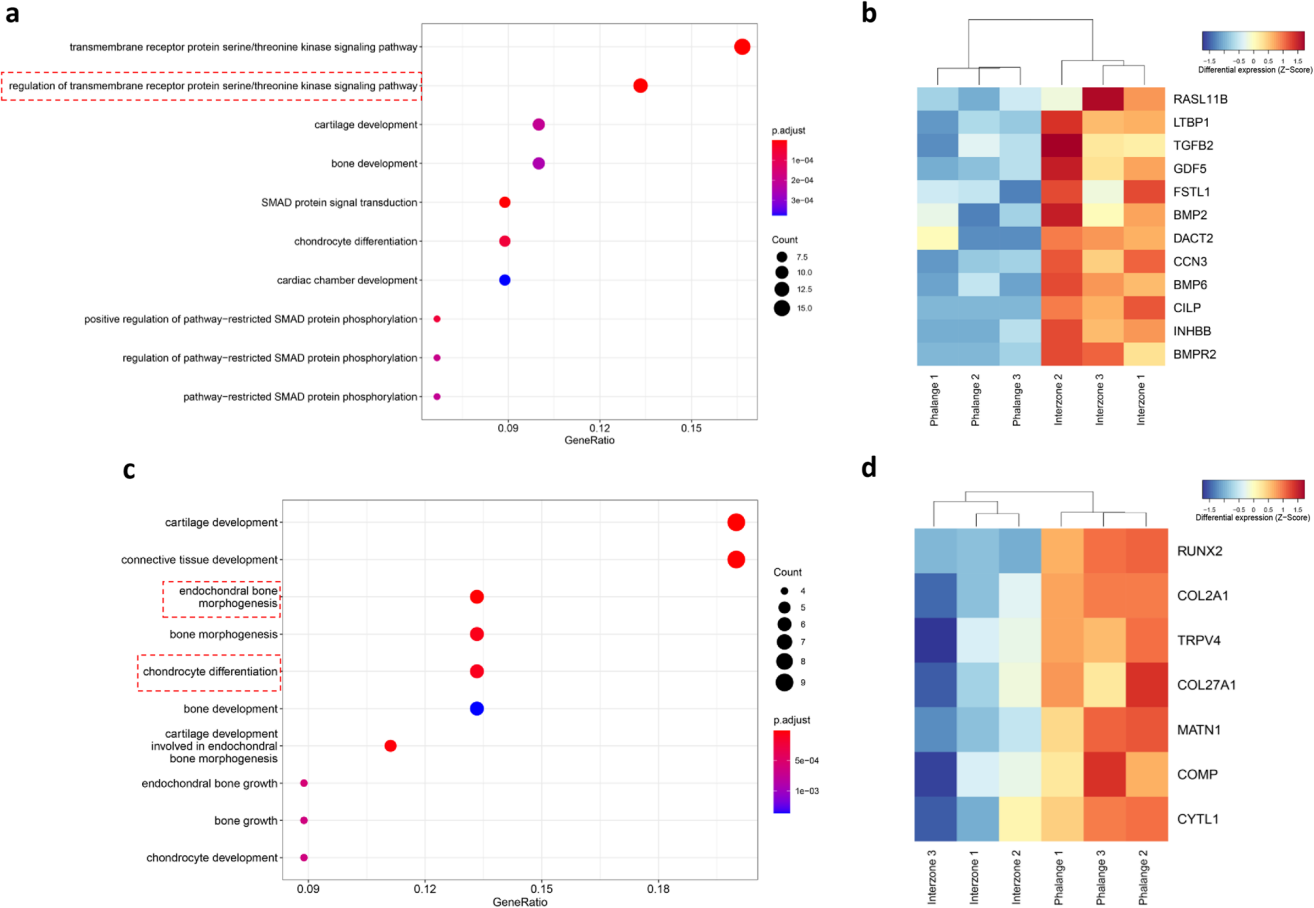
Analysis of the DEGs. (**a**) Pathway enrichment analysis of genes significantly upregulated in interzone as compared to phalange. Color of the dot presents p.adj; size of the dot marks the number of genes involved in the pathway. The red rectangle marks pathway shown as enriched in the analysis of interzone-specific CEs as well as DEGs. (**b**) Heatmap presenting the differences in expression level of genes involved in the pathway: regulation of transmembrane receptor protein serine/threonine kinase signaling. (**c**) Pathway enrichment analysis of phalange upregulated DEGs. The red rectangle highlights pathways identified as enriched in for phalange specific CEs as well as DEGs. Color and size of the dot are as described above. (**d**) Heatmap presenting the expression level between interzone and phalange for genes involved in the pathways: endochondral bone morphogenesis and chondrocyte differentiation, respectively.

To investigate if there was a correlation between gene transcription and CEs, we superimposed our RNA-seq and ChIP-seq data. Pathway enrichment analysis of DEGs showed that genes upregulated in interzone again linked to transmembrane receptor protein serine/threonine kinase signaling (Fig. 6a), in line with our preceding annotation of interzone-exclusive CEs (see above). In particular *RASL11B*, *LTBP1*, *TGFB2*, *GDF5*, *FSTL1*, *BMP2*, *DACT2*, *CCN3*, *BMP6*, *CILP*, *INHBB* and *BMPR2* were found upregulated in interzone as compared to phalange (Fig. 6b). Similarly, analysis of genes upregulated in phalange linked these to chondrocyte differentiation and also endochondral bone morphogenesis (Fig. 6c), which is consistent with functional annotation of phalange-specific CEs. The genes involved it these two latter processes are *RUNX2*, *COL2A1*, *TRPV4*, *COL27A1*, *MATN1*, *COMP* and *CYTL1* (Fig. 6d).

**Figure 7:**
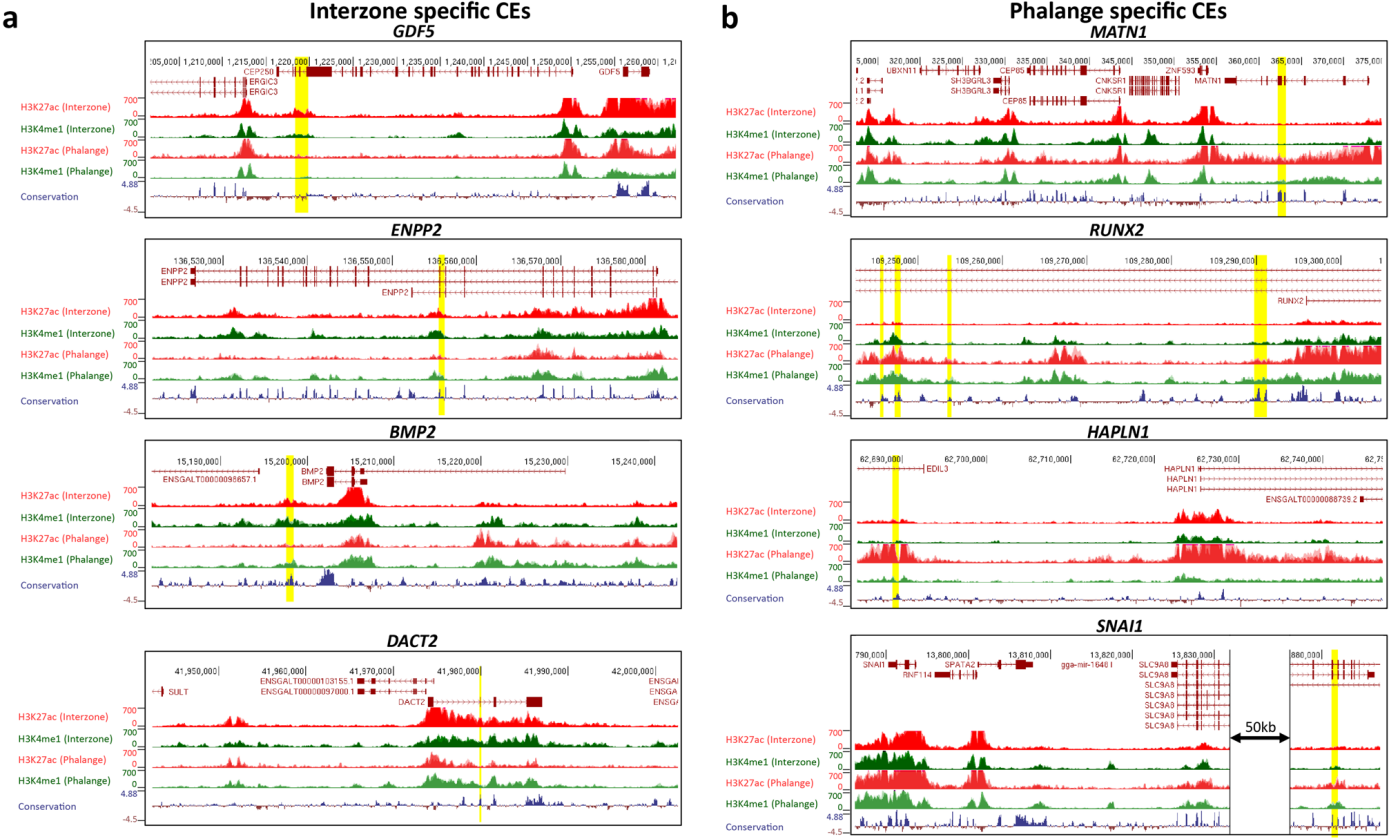
Examples of interzone/phalange-specific CEs that are associated with DEGs. (**a**) CEs associated with selected DEGs upregulated in interzone, for 4 typical loci. As in Fig. 4, the H3K27ac and H3K4me1 enrichment tracks are given in red and green, respectively. The conservation track is marked by blue/brown, and regions marked by yellow present CEs. (**b**) CEs associated with selected DEGs upregulated in phalange, again for 4 typical loci.

Next, in order to identify the CEs that control DEGs upregulated in interzone, the *cis*-regulatory landscapes were characterized by annotation of the TADs encompassing such genes. For this purpose, we used available chicken fibroblast Hi-C data^38^. Next, we extracted CEs located within these TADs, and associated them with DEGs. The CEs were mapped to all DEGs located within the same TAD. Therefore, a CE can be associated with more than one gene, which is in line with studies showing that enhancers can indeed regulate more than one target gene. If the DEGs were located within the region not annotated by any TAD (TADs do not cover the entire chicken genome, as shown by Fishman and co-workers^38^), we associated CEs located ± 1 Mb from the TSS. Using this approach, we identified 486 interzone-specific CEs (Supplementary Table 11; examples of enhancer analysis are given in Fig. 7a).

We then performed the same analysis for phalange-upregulated DEGs. This resulted in identification of 333 phalange-specific CEs (Supplementary Table 12; with examples given in Fig. 7b). Collectively, the integrative analysis of transcriptome data with CEs assignment, and considering interzone vs. phalange signatures, showed that the DEGs involved in cell type-specific processes are regulated by cell-specific CEs.

### CEs regulate skeletal malformation and disease-relevant genes, and are associated with a higher risk of OA

Mutations in genes and CEs have been linked to various limb malformations and skeletal defects^21,23–25^. We applied two types of analysis to screen for CEs that link to molecular etiology of limb disorders in general. First, we assigned our strongly-active CEs to the proximal genes (including relevant respective marker genes and DEGs), and tested whether these genes have previously been associated with limb phenotypes, either in patients (including in syndromes) or mouse models. Analysis of interzone/phalange specific strongly-active CEs showed that these are indeed involved in the regulation of genes linked to joint and phalange abnormalities (Supplementary Table 13a–o).

**Figure 8:**
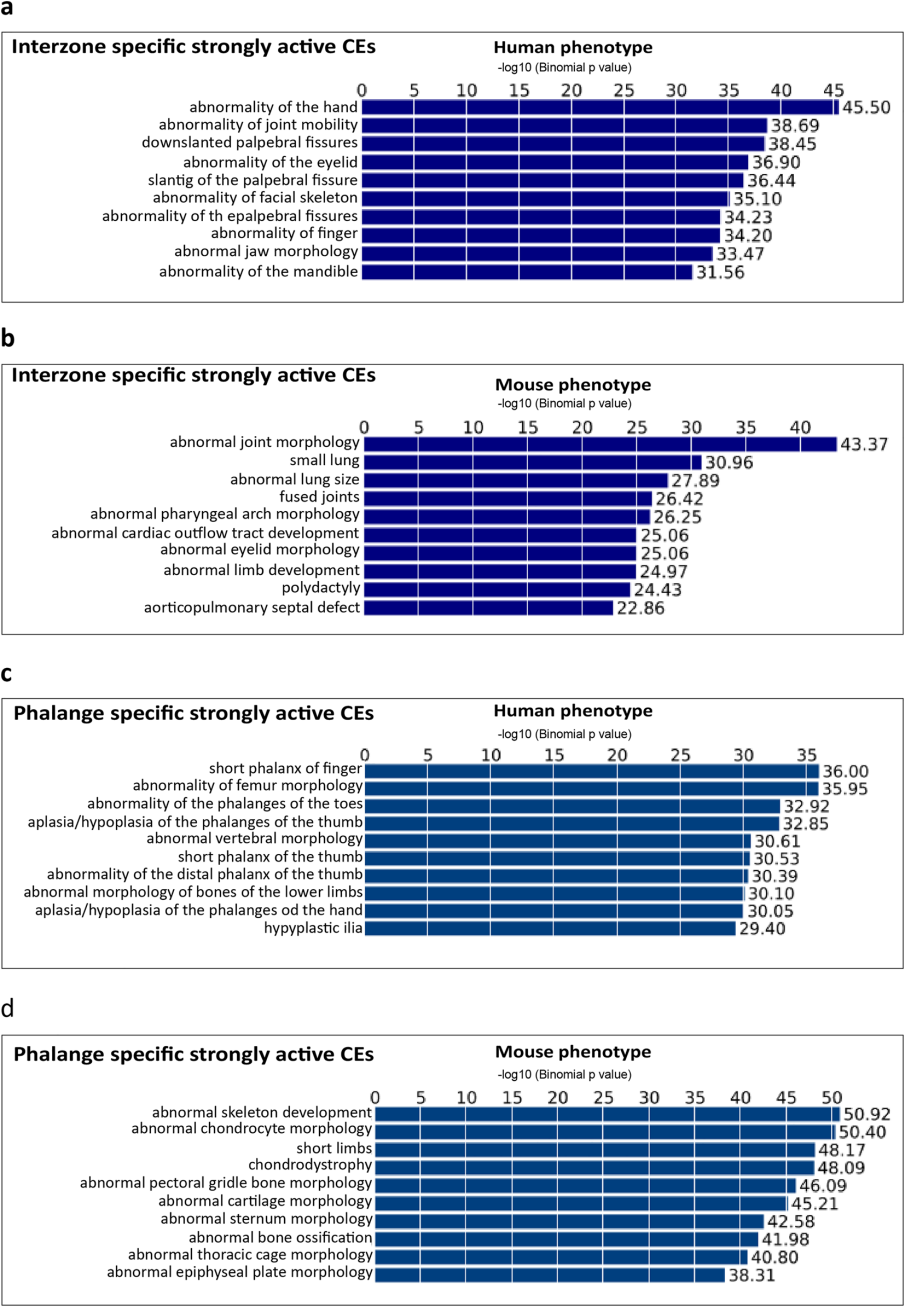
Identified CEs link to synovial joint/phalange disorders. The candidate enhancer regions were assigned to target gene(s) using GREAT, followed by further association with human/mouse phenotypes (for details, see main text). The human (**a**) and mouse phenotypes (**b**) linked to interzone-specific CEs. The human (**c**) and mouse (**d**) phenotypes associated with phalange-specific CEs.

The interzone-specific, strongly-active CEs particularly associate with defective joint mobility in humans (Fig. 8a). For instance, we identified such putative enhancers of *OTX2* and *TGFB2*, which are genes that have been linked to joint laxity (OMIM #610125 and #614816, respectively); candidate CEs of *FLNB*, a gene associated with joint dislocation and carpal fusion (OMIM #150250 and #272460, respectively); we also predicted enhancers of *COL5A1*, a gene linked to joint hypermobility (OMIM #130000) (Supplementary Table 13a). In mice the interzone-specific CEs associate with abnormal joint morphology and fused joints (Fig. 8b; see also Supplementary Table 13b,c). Phalange-specific CEs have in humans been linked to aplasia/hypoplasia of the phalanges, short phalanges, and abnormality of the phalanges of the toe (Fig. 8c). For example, we identified putative enhancers of *BMPR1B* and *IHH* (Supplementary Table 13d; both genes are associated with brachydactyly type-A, OMIM #112500), and candidate enhancers of *RUNX2,* a gene linked to cleidocranial dysplasia, with brachydactyly (OMIM #119600) (Supplementary Table 13f). The phalange-specific CEs have also been linked to abnormal chondrocyte and cartilage morphology, chondrodystrophy, abnormal bone ossification and short limbs in mice (Fig. 8d; see also Supplementary Table 13j–o).

Next, we screened the GWAS catalog (NHGRI-EBI^39^), which resulted in identification of 3263 single-nucleotide polymorphisms (SNPs) within CEs, with 232 of these linking to skeleton-related traits (Supplementary Table 14). For instance, we identified single-nucleotide variations (SNVs) within CEs that have been associated with OA-relevant genes, such as for *ALDH1A2* (rs4775006, P-value 8 × 10^–10^)^40,41^ and *WWP2* (rs34195470, p-value 3 × 10^–13^)^5,42^. Additionally, we identified SNVs associated with increased risk of OA, which are located within CEs that map to *LRIG3* (rs79056043, P-value 1 × 10^–9^), *CRADD* (rs7953280, P-value 5 × 10^–12^) and *ROCR* (rs8067763, P-value 2 × 10^–9^)^5,43^.

We then checked whether the SNPs located in CEs may affect the TF motifs. For this, we used the motifbreakR package, which provides scores for each SNP, both for reference and alternative allele, indicating the importance of the studied TF motifs^44^. The bigger the difference between these two alleles, the stronger motifbreakR predicts a variant effect on the analyzed TF motif. Scores below 0.4 are marked as neutral, < 0.7 as weak, and > 0.7 as strong. Using motifbreakR, we annotated 75% of SNPs located in CEs, out of which three-quarters are predicted to have at least one strong effect on any TF binding motif (Supplementary Tables 15 and 16). Similar proportions were observed among variants related to skeletal traits. For example, rs34195470, associated with *WWP2*, may affect *TAL1* and *SRY* binding motifs (Supplementary Fig. 8). rs4775006 linked to *ALDH1A2* may damage the binding site in *VDR* (Supplementary Fig. 9). Also, rs8067763 associated with *ROCR*, may disrupt the *MECOM* motif (Supplementary Fig. 10). Altogether, the analysis of the tissue-specific CEs showed the association with either synovial joint or phalange congenital abnormalities, which affect their function, as well as the identified CEs of genes relevant to joint degenerative disorders, in particular OA.

## Discussion

Transcriptome analysis of joint interzone gained attention in recent years^14,18,45^, but unlike in other fields, relatively little is known about the *cis*-regulatory elements involved in the establishment of interzone during limb development. Here, we carried out an integrative analysis of transcriptomic and epigenetic data, and subsequently generated a ChIP-seq based CE atlas, for separated interzone and phalange, respectively. For this, we optimized a fast-dissection protocol for careful collection of joint interzone samples. Using both RNA-seq and validation RT-qPCR with selected markers, we showed that such collected interzones have significantly higher expression of *GDF5*, *ENPP2* and *ERG* as compared to adjacent phalange. At the same time, we validated the dissection protocol for collecting interzone cells, for using these in genome-wide experiments that require high numbers of input cells. This optimization of sample collection and separation permitted for the identification of CEs, and correlation of the latter with digit/joint formation.

Functional annotation of the CEs illustrates that strongly-active CE regions enriched in both H3K27ac and H3K4me1 are associated with genes important for cartilage/skeletal development, unlike the CEs enriched in one of the two H3 marks only. Also, Cheung et al.^46^ using differentiated chondrocytes showed that strongly-active CEs identified by ChromHMM are liked to genes pivotal for regulation of the cell-type specific processes, like during chondrogenesis and in cartilage function. In contrast and similarly to our findings, active and poised CEs from differentiated chondrocytes have been associated with more general GO terms. The H3 signature determinations, just as often used extra ATAC-seq or DNase-seq results, likely still miss CEs in general or CEs that are active in the respective cells during earlier of later stages of joint interzone formation. However, to our knowledge this is the first time H3-signatures for these difficult-to-get cell populations in the joint interzone are obtained and provide an important starting point for subsequent studies in the field.

Analysis of TF motifs located in strongly-active CEs shows that these regions are enriched in motifs crucial for both synovial joint and phalange development. Moreover, these CEs are enriched for the PITX1 motif, a TF broadly associated with limb enhancers and involved in establishment of hindlimb identity^34^. Therefore, these CEs might play role in establishment of the hindlimb identity. We also identify CEs unique for interzone/phalange linked to specific biological processes, which correlate with the pathway enrichment analysis of DEGs.

One of the pathways enriched in upregulated genes and CEs in interzones is transmembrane receptor protein serine/threonine kinase signaling, i.e. TGFβ/BMP family signaling. This signaling system has been well-characterized in the process of chondrogenic differentiation^47–50^, but is still not well characterized in joint interzones. Suppression of BMP activity in the interzone region is essential for normal joint development^11,51^. In contrast, *GDF5* is expressed at high level in the interzone region, and *GDF5-null* mutations result in joint defects^52^. In our study we confirm in an alternative way previously described enhancers of *GDF5*, and identify novel CEs of *GDF5*. Another example of a BMP-upregulated gene in the interzone region, consistent with reported in situ RNA-hybridization, is *BMP2*^53^. *BMP2* is involved in joint maturation; its genetic inactivation in synovial joint forming cells results in changes in extracellular matrix and also shape of the meniscus^54^. Interestingly, within the genomic regulatory landscape of *BMP2*, we identified several CEs likely to be active in the interzone, but this will require further investigation. We also documented a change of enhancer state between strongly-active and poised enhancers, which enabled us to identify CEs associated with unique biological processes. Some of these processes correlate with analysis of interzone/phalange-specific CEs and pathway enrichment analysis of certain DEGs.

When considering changes in gene expression causal for limb malformation, it becomes necessary to also include studies of enhancers, which may drive misexpression of disease-causing genes. We show that many CEs associate with genes important for normal development as well as etiology of both synovial joints and phalanges. For instance, in our study we identified CEs of *GDF5* (as shown by Chen and co-workers^28^) to be active in the interzone region. Mutations in *GDF5* lead to joint malformations, and in a genomic region encompassing *GDF5* these experimentally tested enhancers have been linked to higher risk of OA^28,55–57^. We also characterized interzone-specific CEs of the *BMP2* locus. Importantly, *BMP2* conditional knock-out mice develop progressive OA in the knees^54^. Another set of gene-linked enhancers in joint abnormalities are CEs for *FSTL1*, associated with rheumatoid arthritis^58^. We also identified CEs linked to genes (e.g., *OTX2*, *TGFB2*, *COL5A1*) associated with defects in joint mobility.

We used the GWAS catalog NHGRI-EBI to identify SNVs located within CEs and that are associated with higher risk of OA. This yields CEs linked to the genes previously described in OA, such as *ALDH1A2* and *WWP2*. Next, we showed that SNPs located in these CEs disrupts binding motifs for TFs. In addition to interzone CEs, we also characterized several phalange CEs linked to the genes important in chondrocyte-related disorders. For instance, we identified CEs associated with *RUNX2* and *IHH*, both (also) pivotal genes in the molecular etiology of limb malformation, including brachydactyly^59,60^. Altogether, this illustrates that our CE atlas provides information on association of CEs with already existing gene-to-disease correlations. This will be helpful in studies of possible variations in genomic regions in patients without mutations in the protein-coding genes.

We also discovered that *BMPR2* was co-expressed with *GDF5* in interzones. Therefore, it is possible that BMP signaling in the interzone region is prevented at the intracellular level, or BMP ligands play a dual function, which is mutually exclusive in the interzone cells and chondrocytes. Both *GDF5* and *BMPR2* are upregulated in chondrocyte dedifferentiation in vitro^61^, which may support the hypothesis that interzone originates from de-differentiated chondrocytes^9^.

WNT signaling is crucial in the formation of joint interzone. We confirmed that *DACT2* (encoding an intracellular WNT beta-catenin dependent pathway inhibitor) was significantly upregulated in interzone as compared to phalange, consistent with reported in situ RNA-hybridization^62^. This shows again the complexity of WNT signaling regulation during joint formation. Within the genomic regulatory landscape of *DACT2* we identify several CEs that are likely active in the interzone. However, the more detailed characterization of these *DACT2* enhancers requires additional studies.

Our work identified several genes from the TGFβ family ligands or other system components upregulated in interzone, for instance *TGFB2*, *LTBP1* and *INHBB.* Several studies showed antagonistic action of TGFβ/Activin-Nodal pathways on BMP signaling in several cell types^63,64^. Therefore, it is tempting to hypothesize that the upregulation of TGFβ family system components in interzone may have effects on attenuation and/or inhibition of BMP signaling within prospective sites of synovial joint formation.

## Materials and methods

### Tissue collection

All vertebrate animal experiments (with chick early embryos) were carried out in accordance with the relevant guidelines as applied and approved by the Ethical Committee at the Medical University in Lublin, where this work was performed, and also comply with the European regulations (directive 2010/63/EU). The tissue collection was performed on the chicken embryos until 7.5 days post fertilization which is exempt from the Ethical Committee Approval. Chick White Leghorn embryonated eggs were incubated at 38.5 °C in fixed humidity for 7.5 days, followed by evaluation of developmental stage based on the Hamilton Hamburger classification (HH), using a Zeiss Stereo Discovery V8 microscope equipped with 0.63 × Plan Apo S Objective Lens. Selected embryos at HH32 were sacrificed for tissue microdissection. The joint interzones and adjacent phalange samples were microdissected from hindlimb digit-3 using Dumont No.5 forceps (tip dimensions: 0.005 × 0.025 mm).

### RNA extraction

Total RNA was extracted and prepared using Syngen Tissue RNA Kit followed by DNA digestion with QIAGEN RNase-free DNase Set. The RNA quality was validated on 1% agarose gel (for RT-qPCR) or on Agilent 2100 Bioanalyzer system with RNA 6000 Nano Assay (for RNA-seq). All latter samples had a RIN value > 9.0.

### RT-qPCR

Three independently extracted RNAs from both interzone and phalange were reverse transcribed to cDNA using Invitrogen™ SuperScript™ IV Reverse Transcriptase and Oligo(dT) primer. The qPCR was performed using PowerUp™ SYBR™ Green Master Mix II on LightCycler® 480 Instrument II. Gene expression was normalized to expression of *GAPDH*. Statistics were computed using Mann–Whitney–Wilcoxon Test. The list of used primers is given in Supplementary Table 17.

### RNA-seq library preparation

The RNA-seq libraries were prepared as previously described^65^. The 6 samples (3 of interzone, 3 of phalange biological replicates) were prepared with the Smart-seq2 method. In brief, poly(A)-RNA was reverse transcribed using oligo(dT) primers. Template switching by reverse transcriptase was achieved by using a LNA-containing TSO oligonucleotide. The reverse-transcribed cDNA was pre-amplified with primers for 18 cycles, followed by clean-up. Tagmentation was performed on 500 pg of the pre-amplified cDNA with Tn5 followed by gap repair. The tagmented library was extended with Illumina adaptor sequences by PCR for 14 cycles and purified. The resulting sequencing library was measured on Bioanalyzer and equimolar amounts loaded onto a flowcell and sequenced according to the Illumina TruSeq v3 protocol on the HiSeq2500, with a single- read 50 bp and dual 9 bp indices.

### RNA-seq data analysis

The fastq files were checked for quality using FastQC (https://www.bioinformatics.babraham.ac.uk/projects/fastqc/) followed by removal of adapters using Trimmomatic^66^. Further, reads were mapped to the *Gallus gallus* 6.0 reference genome using STAR with default parameters^67^. Gene expression values were called using featureCounts with Ensemble release 104 annotation^68^. The differential data analysis has been performed with DEseq2^69^, and heatmaps have been created in R environment for statistical computing. The expression of DEGs were compared to aviable scRNAseq data from Bian et al.^18^, using the web application: cahanlab.org/resources/joint_ontogeny.

### Chromatin-immunoprecipitation (ChIP)

For each sample, either 100 interzones or phalanges were dissected from the 3rd hindlimb digit and pooled together. Further, tissues were dissociated for 3 h at 37 °C using 2.4% Collagenase-II (Gibco™) resuspended in DMEM/high-glucose medium containing 10% fetal bovine serum (FBS). Cells were passed through a 40-μm cell strainer (BD Falcon) and then counted using a hemocytometer. 10^6^ cells were cross-linked using 1% formaldehyde at room temperature (RT, 24 °C) for 9 min. Fixation was quenched with ice-cold 0.125 M glycine for 5 min at 4 °C. To remove excess formaldehyde, two rounds of centrifugation of the cells followed by resuspension in ice-cold PBS were carried out. Next, the cell nuclei were isolated using ice-cold nuclei extraction buffer (NEB) containing 10 mM Tris–HCl pH 7.5, 150 mM NaCl, 1 mM EDTA, 1% IGEPAL® CA-630 and complete protease inhibitors (Roche). Subsequently, the nuclei were resuspended in SDS-containing lysis buffer (50 mM Tris–HCl pH 8.0, 10 mM EDTA and 1% SDS), and chromatin was sheard obtaining the average size of 150 bp in AFA Fiber Pre-Slit Snap-Cap (130 µl) microtube using a S220 Focused-ultrasonicator.

For ChIP, 500 ng of sonicated chromatin was immunoprecipitated with 7.5 μg of anti-H3K27ac antibody (Active Motif, Cat. No. 39133) or anti-H3K4me1 (61781). Input sample was collected prior to immunoprecipitation reaction. Chromatin pre-cleaning incubation with protein-A and protein-G agarose beads (Millipore) was carried out in immunoprecipitation buffer (50 mM Tris–HCl pH 8.0, 0.15 M NaCl, 1 mM EDTA pH 8.0, 1% Triton X-100 and 0.1% sodium deoxycholate) for 3 h at 4 °C while rotating. In parallel, the antibodies were incubated with previously blocked A- and G-agarose beads (Millipore) also for 3 h at 4 °C, again while rotating.

After pre-cleaning, the chromatin was mixed with the pre-bound antibodies with A- and G-agarose beads and incubated overnight (O/N) at 4 °C, rotating. The next day, multiple rounds of washes of the beads were conducted. Each wash was carried for 10 min at 4 °C while rotating. The beads were washed once with RIPA-150 buffer (50 mM Tris–HCl pH 8.0, 0.15 M NaCl, 1 mM EDTA pH 8.0, 0.1% SDS, 1% Triton X-100, 0.1% sodium deoxycholate), twice with RIPA-500 (50 mM Tris–HCl pH8.0, 0.5 M NaCl, 1 mM EDTA pH8.0, 0.1% SDS, 1% Triton X-100, 0.1% sodium deoxycholate), once with RIPA-LiCl (50 mM Tris–HCl pH 8.0, 1 mM EDTA pH 8.0, 1% Nonidet-P40, 0.7% sodium deoxycholate, 0.5 M LiCl) and twice in TE buffer (10 mM Tris–HCl pH 8.0, 1 mM EDTA pH 8.0). Subsequently, the chromatin was eluted with 200 μl of fresh elution buffer (1% SDS and 0.1 M NaHCO_3_) followed by addition of 100 μl of TE buffer and 25 μl of 5 M NaCl prior to reverse-crosslinking at 65 °C for 16 h. The next day, chromatin was incubated with 2 μl of Proteinase-K (10 mg/ml) for 1 h at 56 °C, and 2 μl of RNaseA (10 mg/ml) for 45 min at 37 °C, and DNA was further purified using QIAquick PCR Purification Kit (QIAGEN). The size distribution of immunoprecipitated fragments was evaluated using Agilent 2100 Bioanalyzer system with High Sensitivity DNA. Additionally, the DNA-concentration of input and immunoprecipitated samples was measured on Qubit 2.0 Fluorometer (Invitrogen).

### ChIP-sequencing

ChIP-seq libraries were generated as previously described^70^. Shortly, libraries were prepared using QIAseq Ultra Low Input Library Kit (QIAGEN, Hilden, Germany). Briefly, DNA was end-repaired, adenosines were added to the 3′ ends of dsDNA and adapters were ligated (adapters from NEB, Ipswich, MA, USA). Following the adapter ligation, uracil was digested by USER enzyme from NEB (Ipswich, MA, USA) in a loop structure of the adapter. Adapters containing DNA fragments were amplified by PCR using NEB starters (Ipswich MA, USA). Library quality evaluation was done with Agilent 2100 Bioanalyzer using the Agilent DNA High Sensitivity chip (Agilent Technologies, Ltd.) Quantification and quality evaluation of obtained samples were done using Nanodrop spectrophotometer (Thermo Scientific, NanoDrop products, Wilmington, USA), Quantus fluorometer (Promega Corporation, Madison, USA) and 2100 Bioanalyzer (Agilent Technologies, Santa Clara, USA). Mean library size was 300 bp. Libraries were run in the rapid run flow cell and were single-end sequenced (65 bp) on HiSeq 1500 (Illumina, San Diego, CA 92122 USA).

### ChIP-seq data analysis, CEs identification and CE annotation

The quality of raw fastq files were validated using FastQC and adapters were removed using Trimmomatic. Next, reads were mapped to the Gallus gallus 6.0 reference genome using Bowtie 2 with default parameters^71^ and PCR-duplicates were marked and removed using Picard (http://broadinstitute.github.io/picard/). The peaks were called using MACS2 with significance level threshold FDR < 0.05, and normalization to input sample^72^. Further, biological replicates were merged using BEDTools^73^.

The unsupervised clustering of H3K27ac and H3K4me1 peaks was performed using DiffBind (https://bioconductor.org/packages/DiffBind/) with normalized IP samples to input. The experiment-specific lists containing anomalously enriched regions were generated using the GreyListChIP and further removed from datasets.

CEs were identified using in-house script. Briefly, the promoter regions (1 kb ± from TSS) were filtered out from H3K27ac and K3K4me1 dataset and nearby peaks (< 1 kb) were merged with GenomicRanges::reduce(min.gapwidth = 1000)^74^. Further, the conserved CEs were selected using BEDTools intersect followed by merging nearby genomic intervals with GenomicRanges::reduce(min.gapwidth = 1000). The consensus and cell-specific CEs were identified using BEDTools::intersect − f 0.9 − r and BEDTools::intersect − v, respectively. The functional interpretation of CEs was performed using GREAT, with the genomic regions previously lifted to hg38 genome using liftOver (https://genome.ucsc.edu/cgi-bin/hgLiftOver).

Tissue-specific CEs have been mapped to the DEGs using our in-house script. Specifically, we characterized the cis-regulatory landscapes of such genes defined by the borders of TADs. To do this we utilized the annotated TADs from chicken fibroblasts^38^. We identified the CEs located within the TADs encompassing DEGs and further associated them with DEGs. If the gene was located within genomic region not annotated by any TAD we defined cis-regulatory landscape as region ± 1 Mb from the TSS.

The enrichment tracks for H3K4me1 and H3K27ac ChIP-seq data were generated using deepTools^75^. Specifically, bamCoverage with reads per kilobase per million mapped reads (RPKM) per bin normalization was used. The enrichment tracks were visualized by loading to UCSC Genome Browser. The tracks visualized in UCSC Genome Browser were merged for biological replicates using transparent method (https://genome.ucsc.edu/cgi-bin/hgCollection).

### Motif enrichment analysis of CEs

The motif enrichment analysis was preformed using HOMER^33^ tool with function “findMotifsGenome.pl”. The CEs coordinates were adjusted to 1 kb from the center of CE region. The randomly selected regions (1 kb size) from chicken genome as background. A *p* value ≤ 0.01 was considered to select significantly enriched motifs.

### Annotation of CEs to disease-relevant genes, and identification of CEs associated with a higher risk of OA

To annotate CEs to disease-relevant genes and locate SNP within the CEs these genomic regions were lifted to hg38 genome using liftOver. The GREAT was used to associate CEs with genes and retriever human and mouse phenotypes. The GenomicRanges::findOverlaps was used to identify SNPs located within the CEs. To assess whether detected SNPs might damage motifs recognized by Transcription Factors, the motifbreakR tool was used^44^. Output was generated based on: human reference genome hg38, the SNPs were liftover to human reference genome hg38, and dbSNP versioned 155. No upstream filtering was performed except for removing variants with the same rsID. Default settings were used with the maximum p-value for a match to be called or a minimum score threshold set to 10^–4^.

## Supplementary Information

Supplementary Figures.

Supplementary Information 1.

Supplementary Information 2.

Supplementary Information 3.

## Data Availability

The RNA-seq and ChIP-seq data are available under the gene expression omnibus (GEO) Accession Number GSE198819.
