# Supplementary material for: Identification of candidate enhancers controlling the transcriptome during the formation of interphalangeal joints
Source: Sci Rep. 2022 Jul 27;12:12835. doi: 10.1038/s41598-022-16951-4 (PMC9329285; doi:10.1038/s41598-022-16951-4)

# Legends to Supplementary Figures

**Supplementary Figure 1. Analysis of RNA-seq data from isolated joint interzone and adjacent phalange.**

(a) Principal component analysis (PCA) showing separation of analyzed interzone and phalange biological replicates based on the origin of the tissue. (b) Dispersion plot presenting the relationship between dispersion and gene expression. (c) Heatmap of all differentially expressed genes.

**Supplementary Figure 2. Analysis of gene expression of DEGs upregulated in phalange using data from a single cell transcriptional atlas of early synovial joint development.**

(a) A UMAP with annotated clusters SC2_A and SC2_B. The SC2_A was characterized by Bian et. al[18] as a cluster containing cells with high expression of chondrocyte markers. The SC2_B was described as a cluster containing cells with high expression of interzone markers. (b) Analysis of DEGs with significantly higher expression in phalange as compared to interzone.

**Supplementary Figure 3. Analysis of gene expression of DEG upregulated in interzone using data from a single cell transcriptional atlas of early synovial joint development.**

(a) A UMAP with marked clusters SC2_A and SC2_B. The SC2_A and SC2_B contain cells as described in the Supplementary Fig. 2. (b) Analysis of DEGs with significantly higher expression in interzone as compared to phalange.

**Supplementary Figure 4. Analysis of gene expression of DEGs upregulated in either phalange or interzone using data from a single cell transcriptional atlas of early synovial joint development.**

(a) A UMAP with annotated clusters SC1-SC3. The SC2_A and SC2_B contain cells marked by the phalange and interzone markers, respectively. (b) Analysis of DEGs upregulated in interzone. (c) Analysis of DEGs upregulated in phalange.

**Supplementary Figure 5. Functional annotation of the candidate enhancers (CEs) in interzone and phalange.**

The CEs were associated with target genes using GREAT, followed by pathway enrichment analysis. Biological process GO terms associated with active and poised enhancers in interzone and phalange, respectively, are represented.

**Supplementary Figure 6. Validated active enhancers identified in our CE atlas.**

Examples of experimentally validated enhancers with limb activity (collected in Vista Enhancer Browser). H3K27ac enrichment track is colored in red, the H3K4me1 enrichment in green, and the conservation track in blue. The gene structure information is marked in brown. Regions marked by yellow present functionally validated enhancers. Activity of these enhancers is shown by the β-galactosidase (blue) staining present in embryonic regions.

**Supplementary Figure 7. Mutually exclusive strongly-active enhancers in biological processes in interzone and phalange.**

(a) GO terms associated with interzone-exclusive strongly-active enhancers, and strongly-active enhancers in interzone that are poised in phalange, and (b) vice versa.

**Supplementary Figure 8. The rs34195470 is predicted to cause strong motif disturbance of TAL1 and SRY binding sites.**

**Supplementary Figure 9. The rs4775006 is predicted to affect the VDR binding site.**

**Supplementary Figure 10. The rs8067763 is predicted to damage the MECOM binding site.**

Supplementary Figure 1

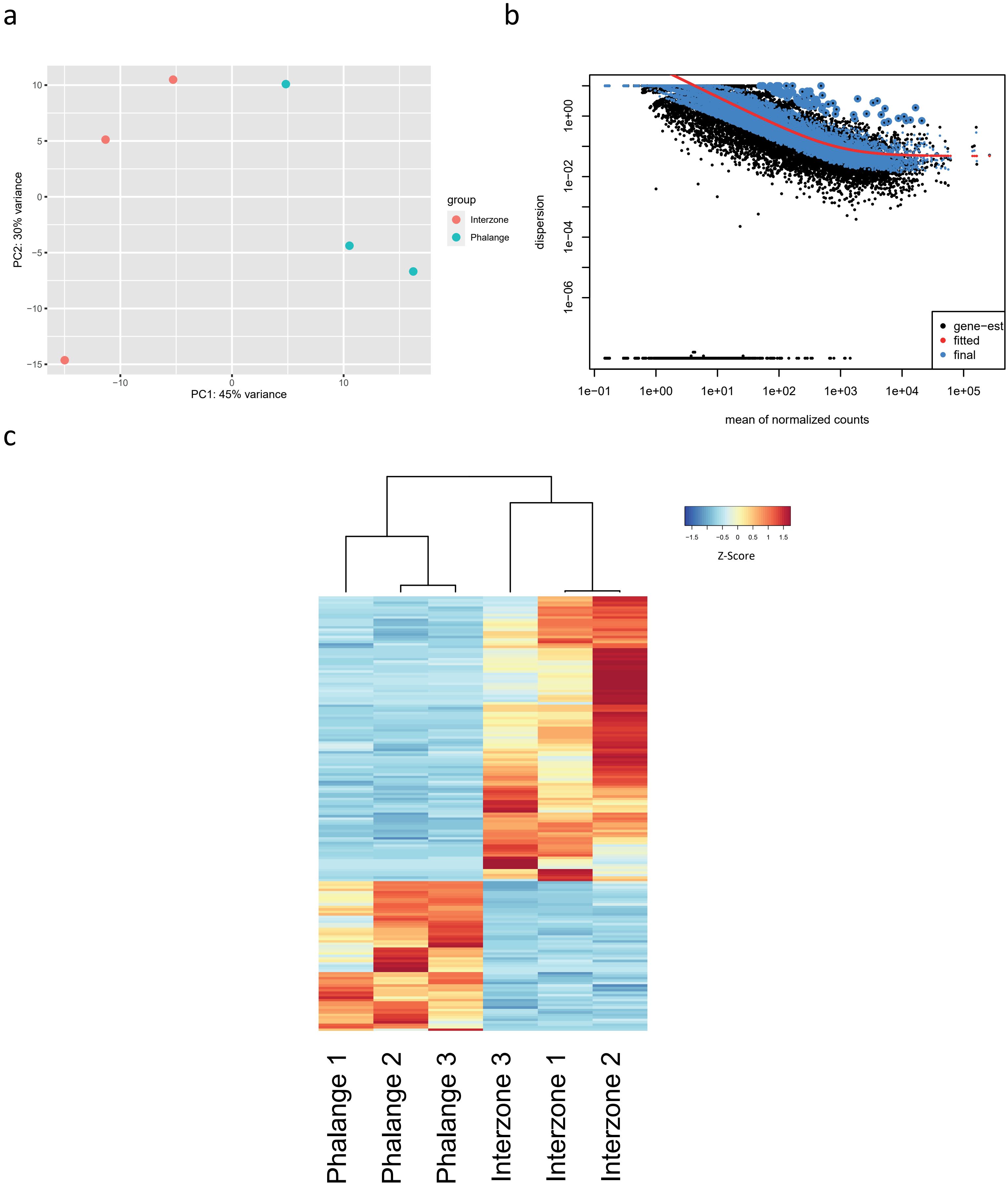

Supplementary Figure 2

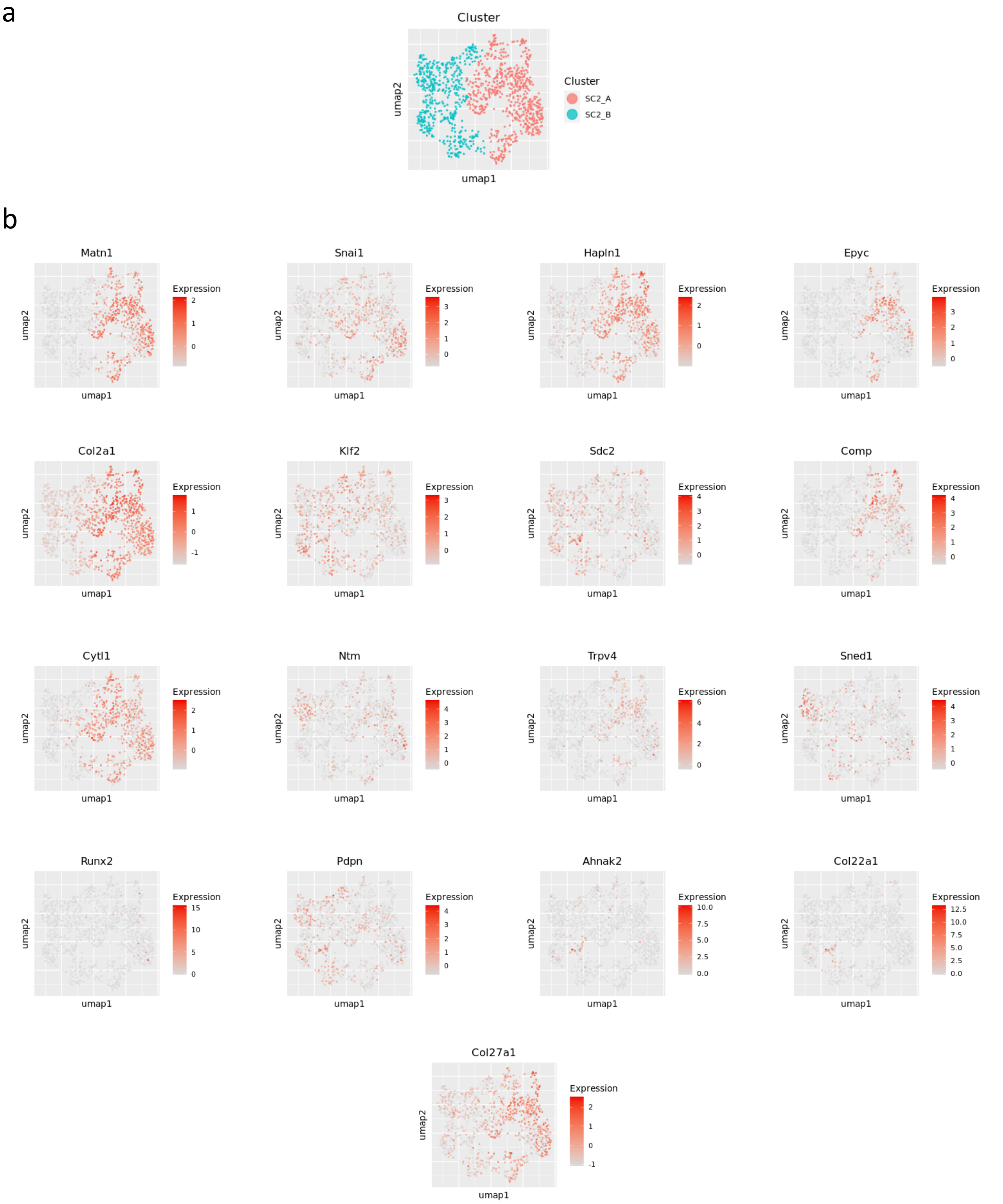

Supplementary Figure 3

a

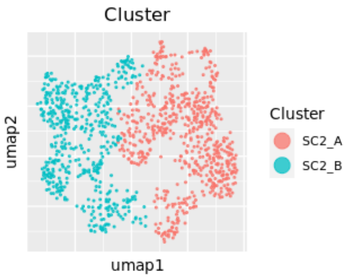


b

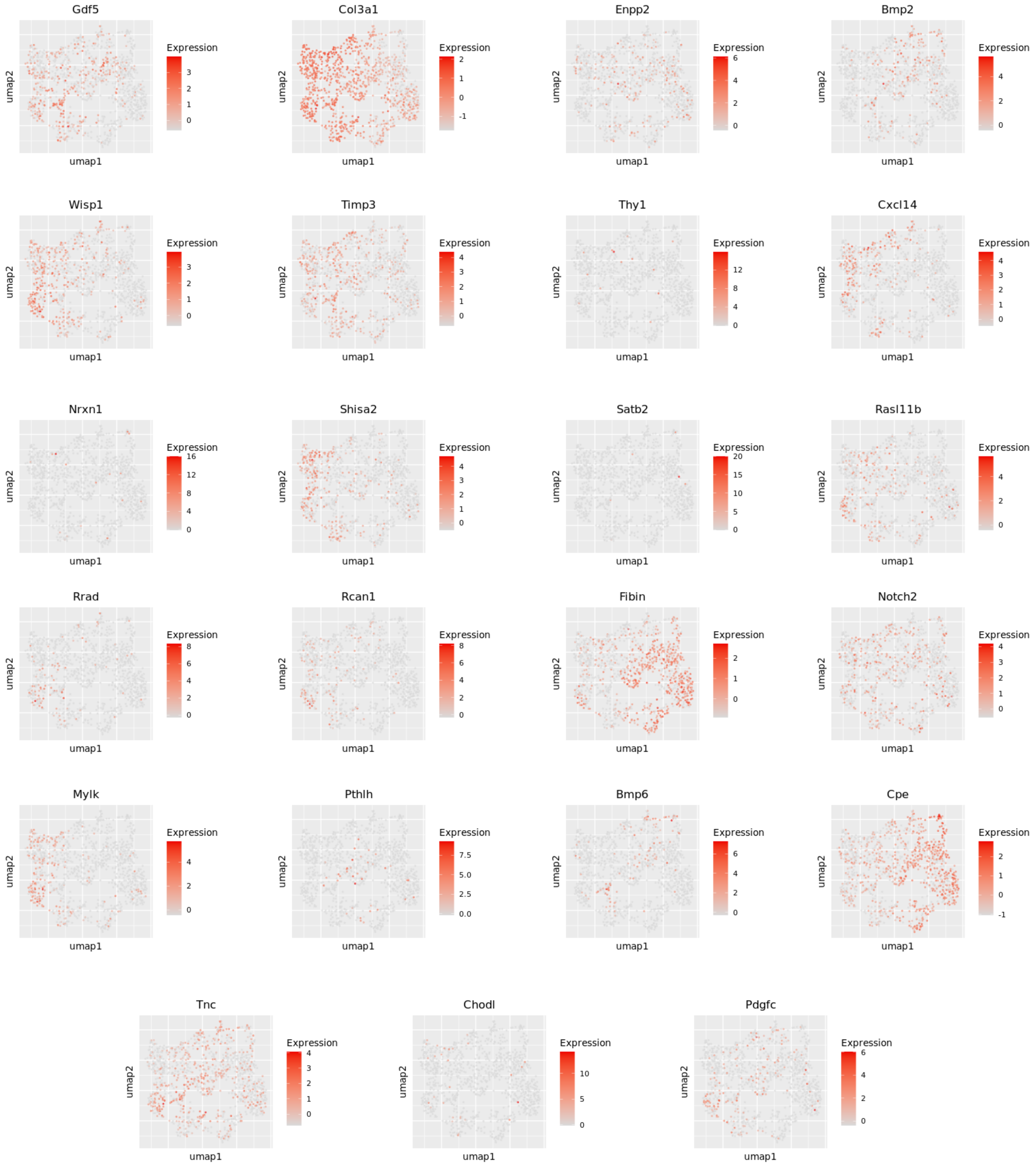

Supplementary Figure 4

a

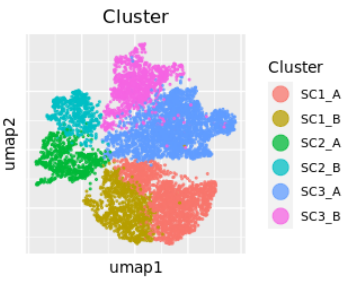


b

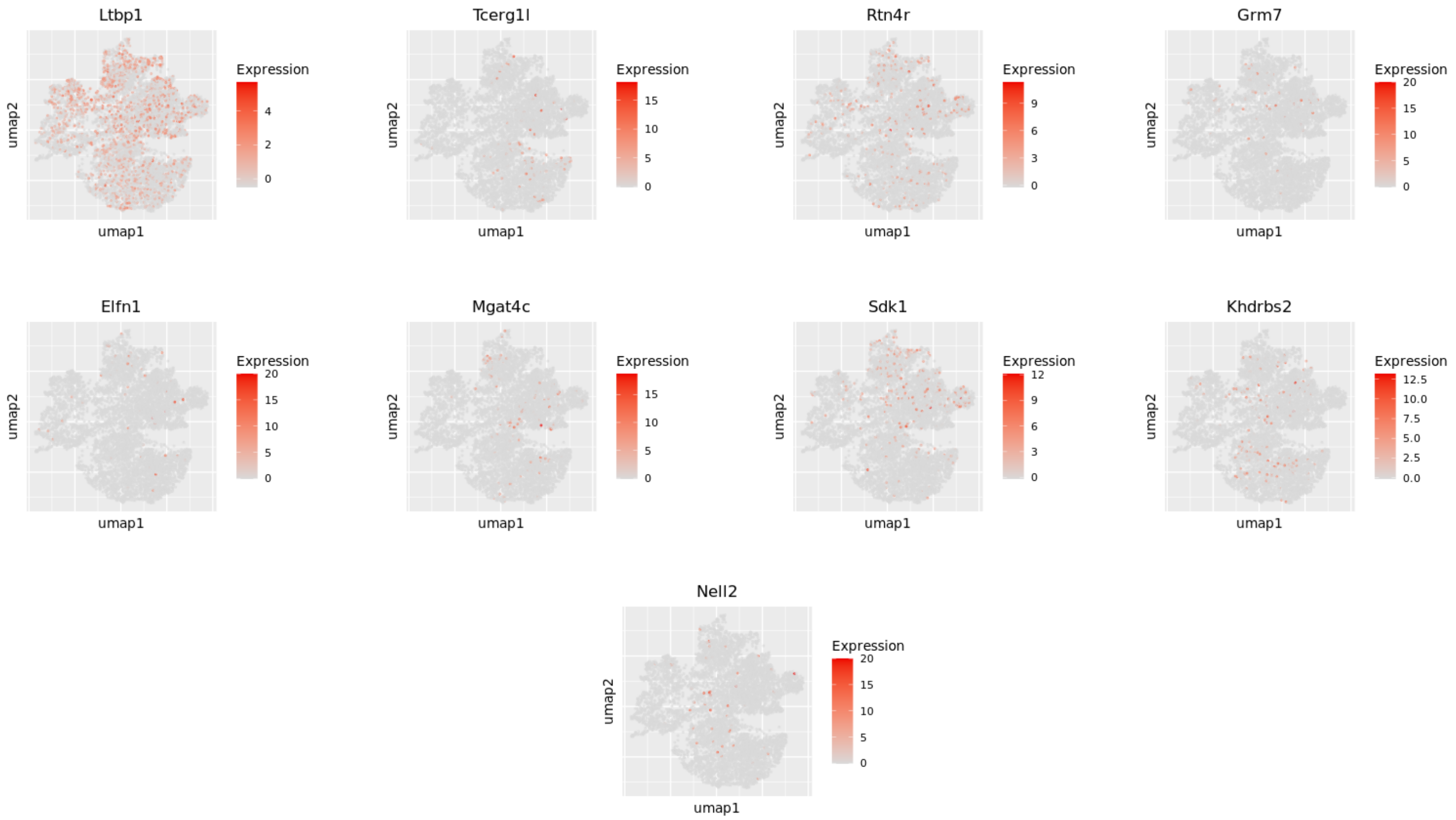


c

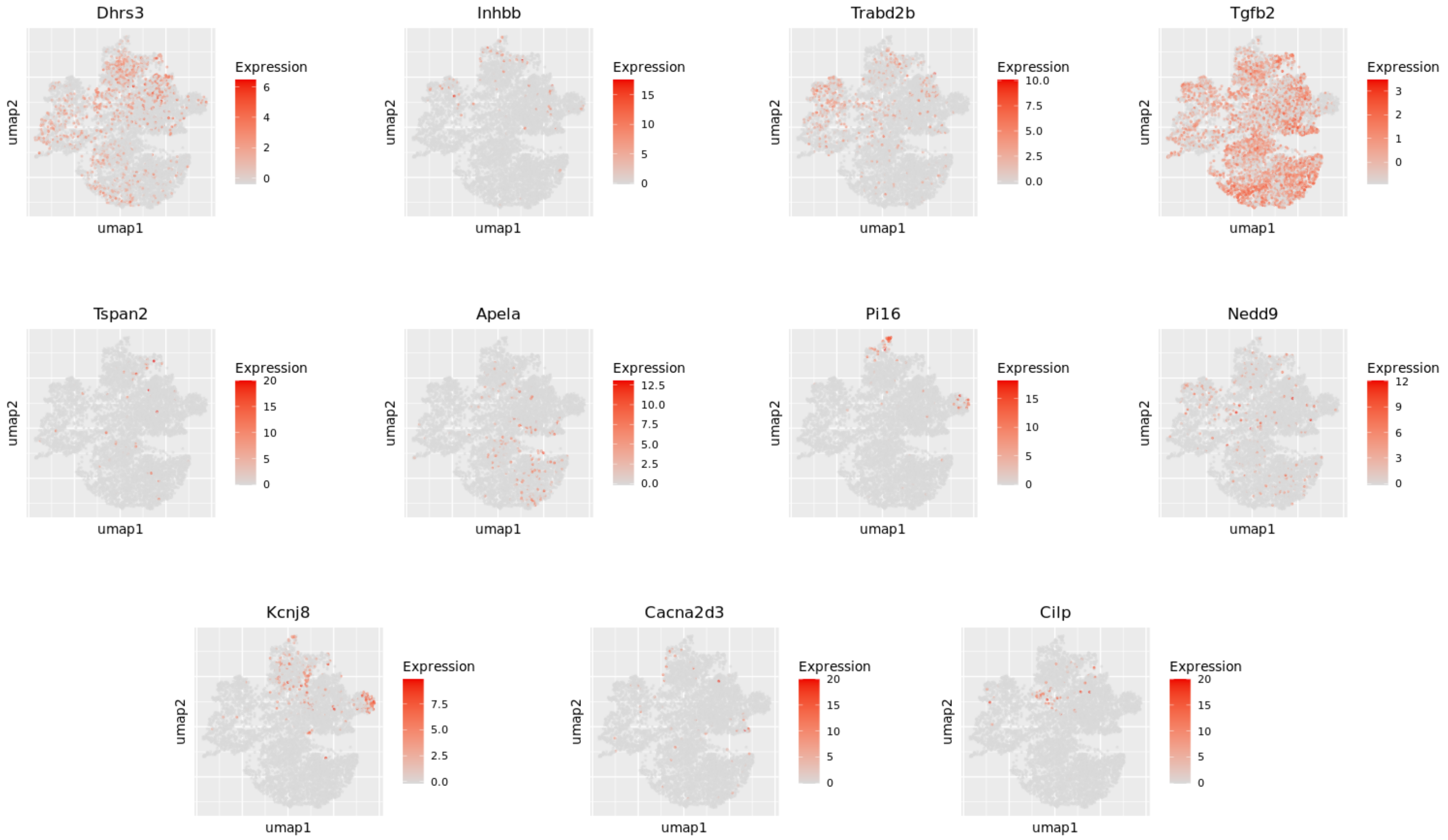

Supplementary Figure 5

# Interzone

## Active enhancers

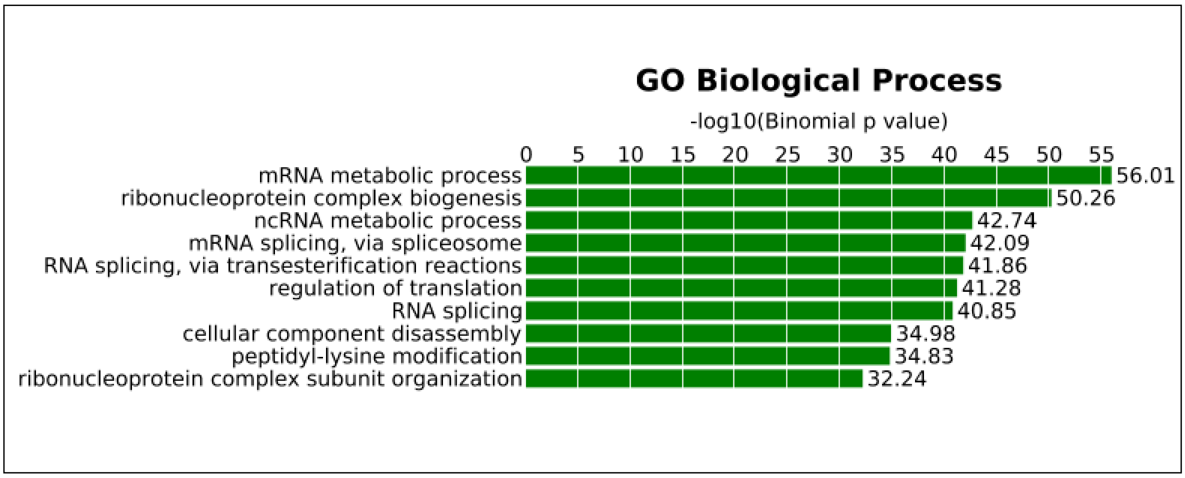


## Poised enhancers

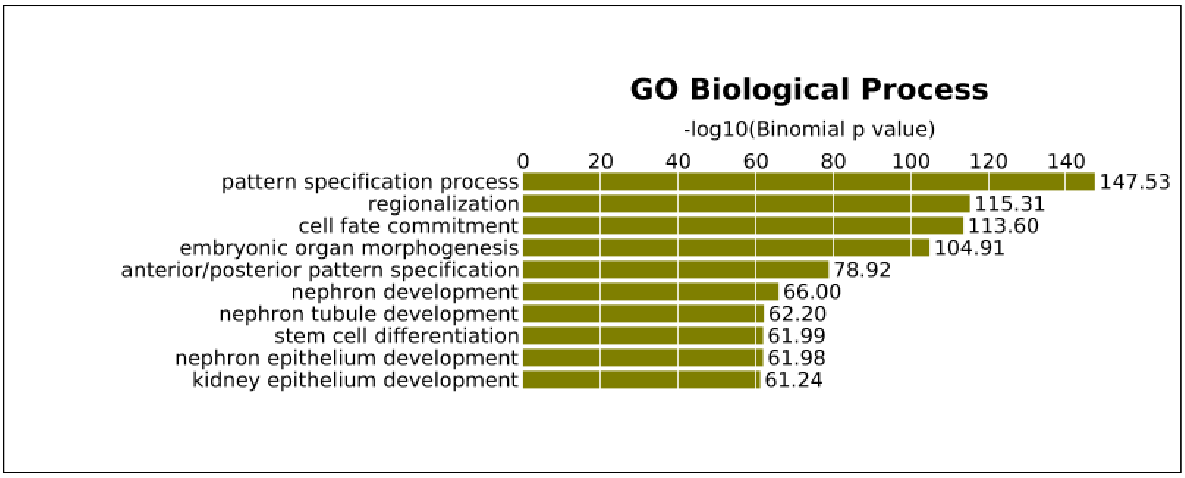


# Phalange

## Active enhancers

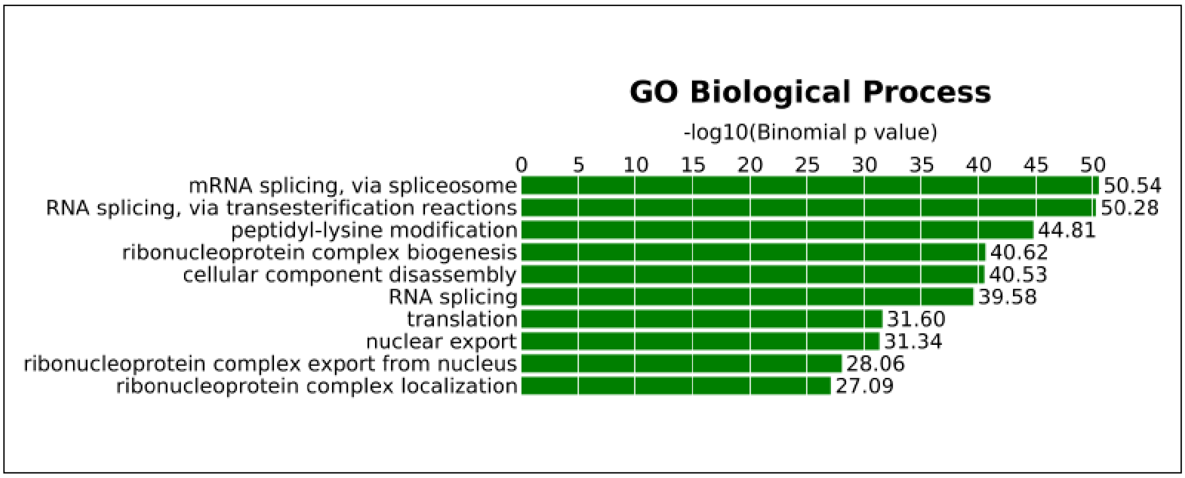


## Poised enhancers

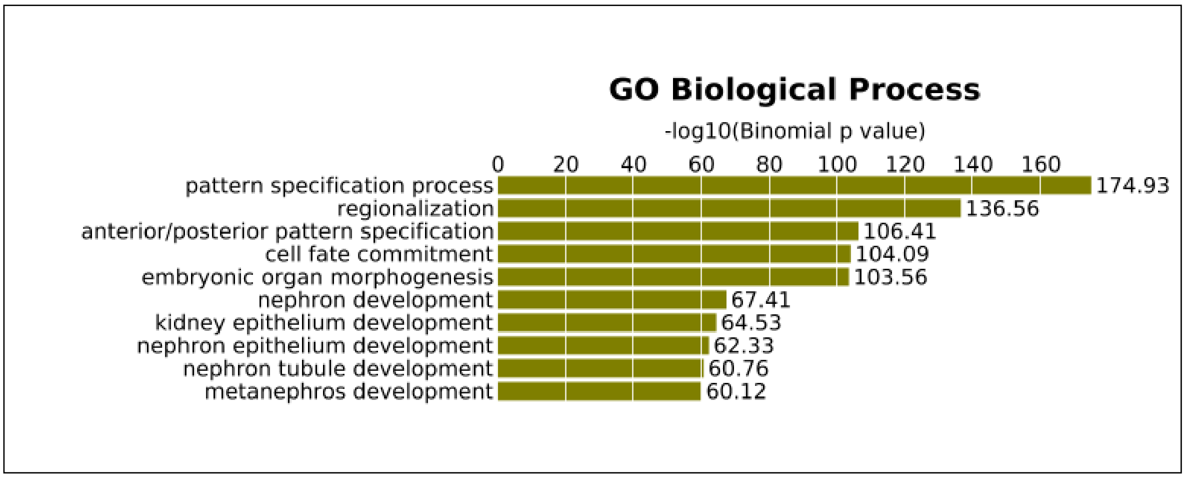

Supplementary Figure 6

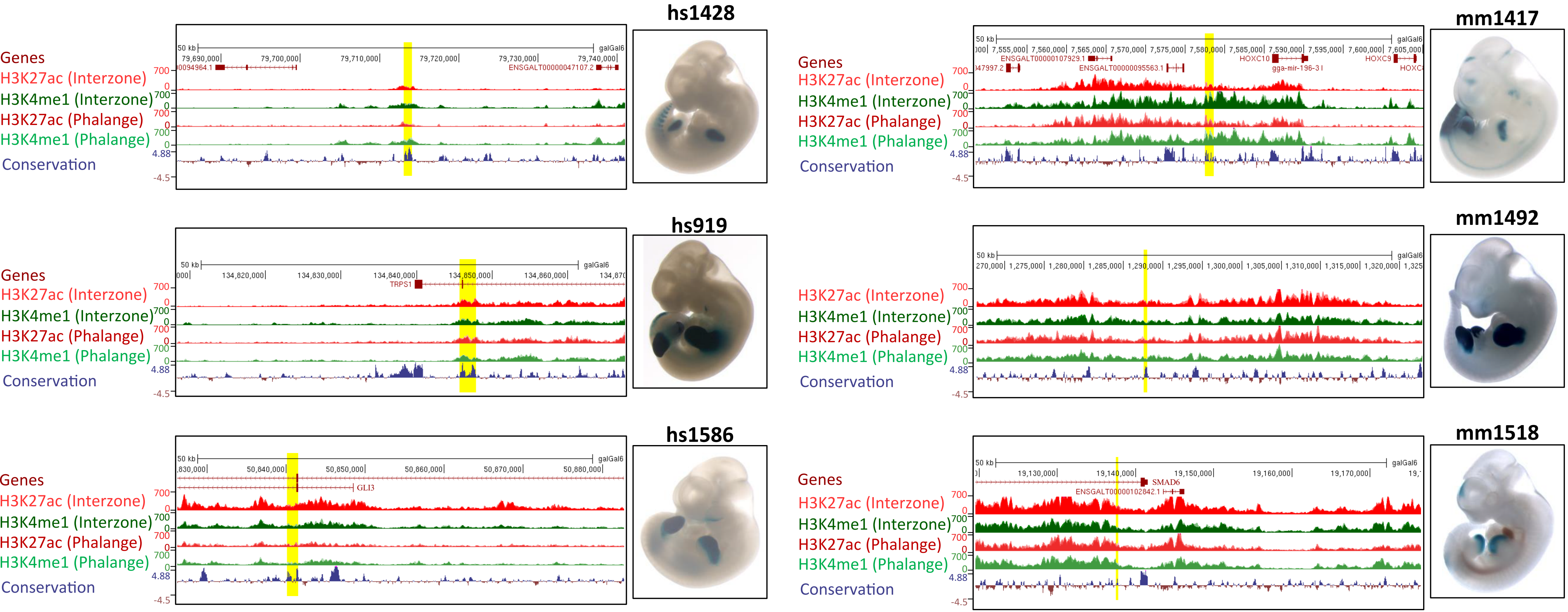

Supplementary Figure 7

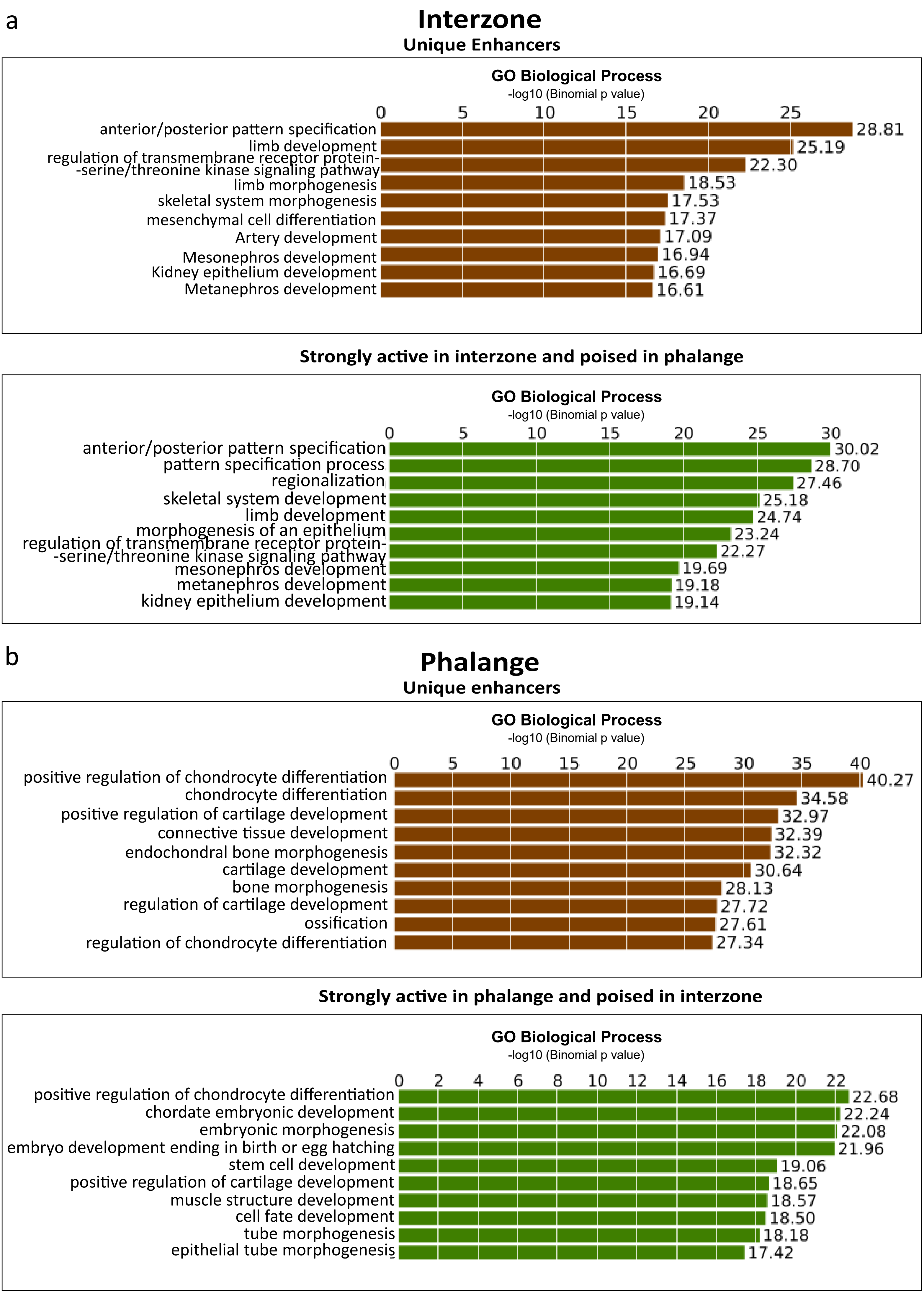

Supplementary Figure 8

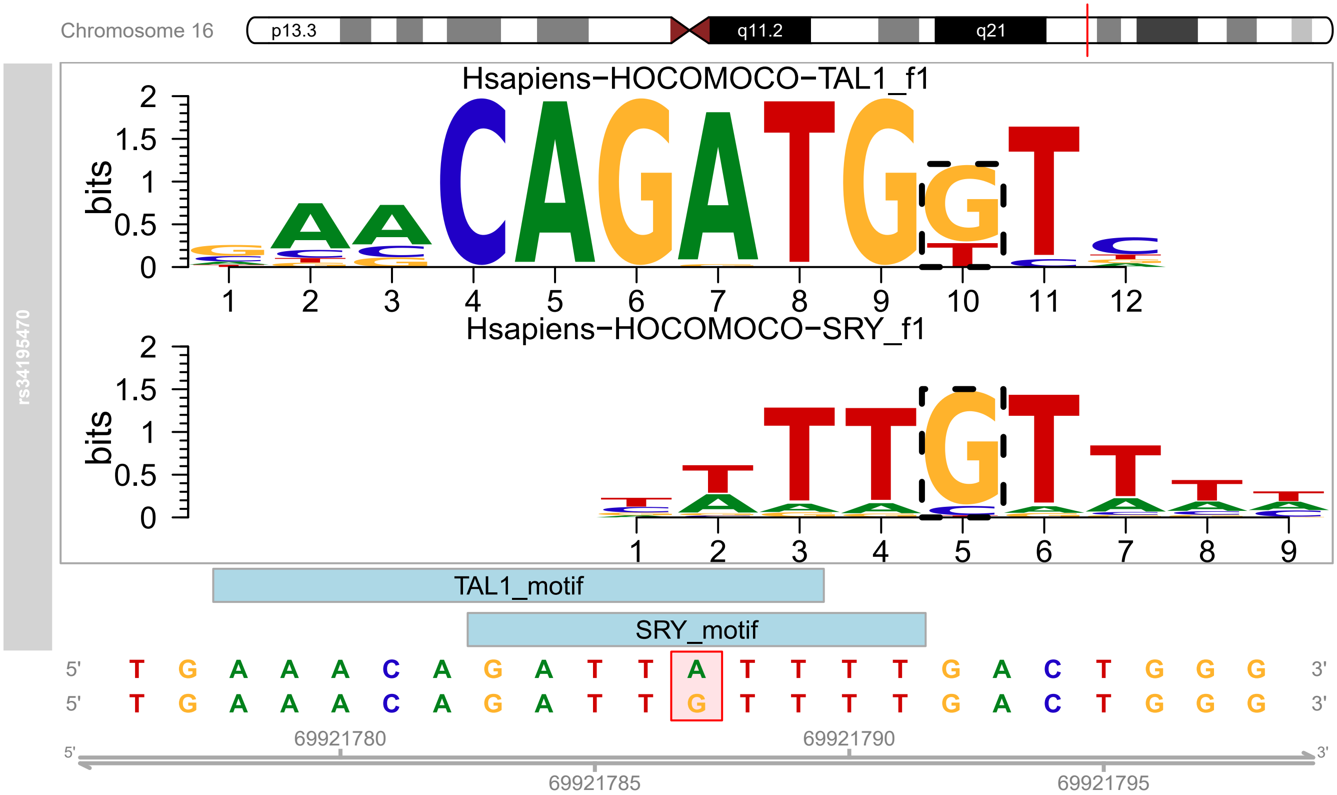

Supplementary Figure 9

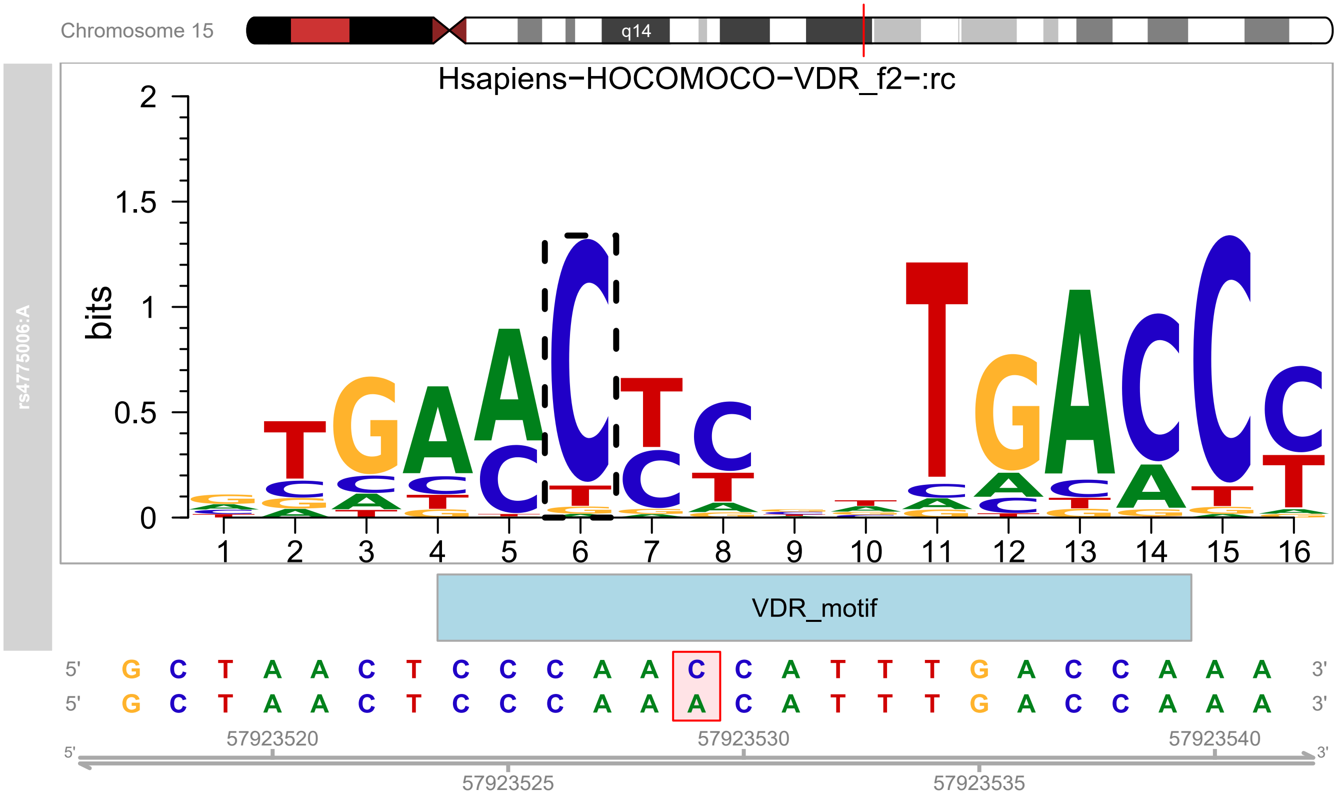

Supplementary Figure 10

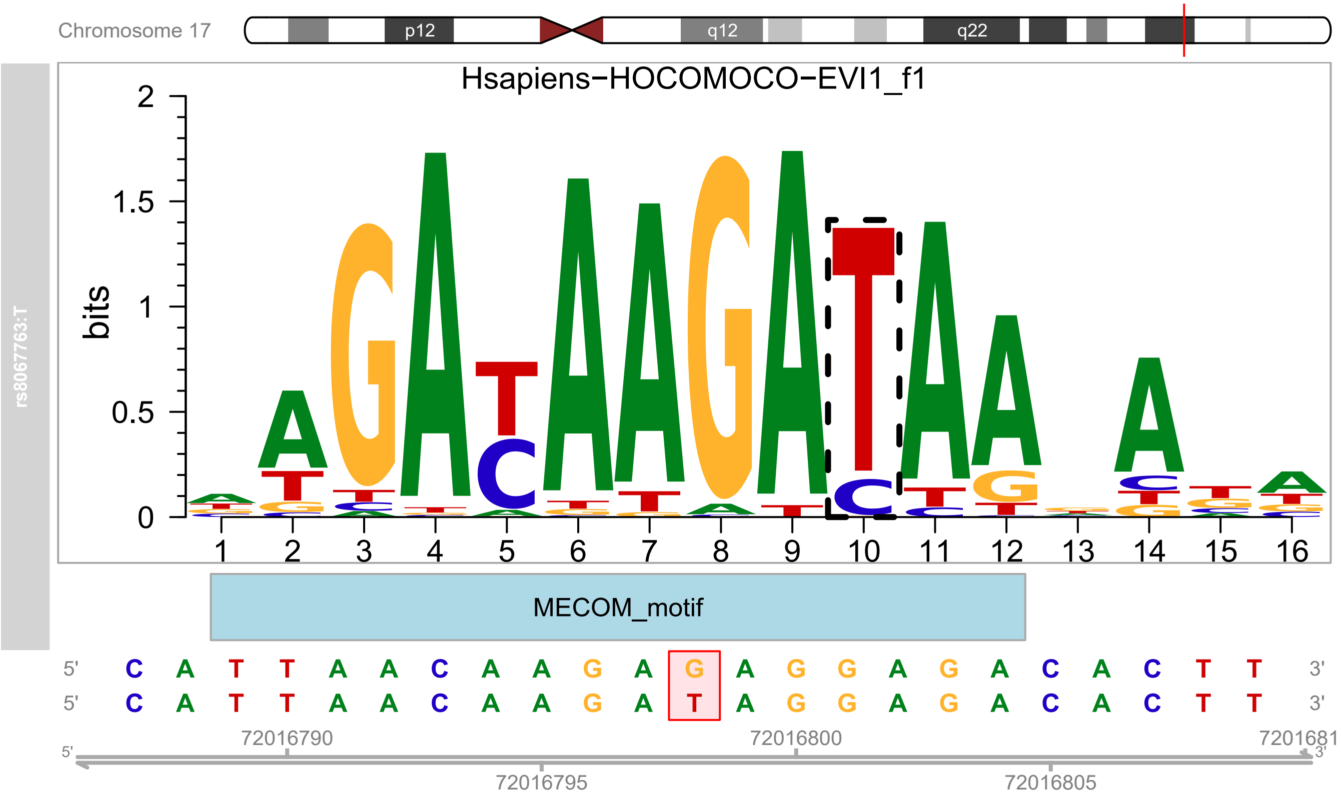

Supplement: Supplementary file 1 — Supplementary Figures. [file 41598_2022_16951_MOESM1_ESM.pdf]
